# Inflammatory arthritis disrupts gut resolution mechanisms, promoting barrier breakdown by *Porphyromonas* *gingivalis*

**DOI:** 10.1172/jci.insight.125191

**Published:** 2019-07-11

**Authors:** Magdalena B. Flak, Romain A. Colas, Estefanía Muñoz-Atienza, Michael A. Curtis, Jesmond Dalli, Costantino Pitzalis

**Affiliations:** 1 Centre for Experimental Medicine & Rheumatology, William Harvey Research Institute, Queen Mary University of London (QMUL), London, United Kingdom.; 2 Lipid Mediator Unit, William Harvey Research Institute, QMUL, London, United Kingdom.; 3 Dental Institute, King’s College London, London, United Kingdom.; 4 Centre for Inflammation and Therapeutic Innovation, QMUL, London, United Kingdom.

**Keywords:** Immunology, Inflammation, Arthritis, Macrophages, Molecular pathology

## Abstract

Rheumatoid arthritis is linked with altered host immune responses and severe joint destruction. Recent evidence suggests that loss of gut homeostasis and barrier breach by pathobionts, including *Porphyromonas gingivalis*, may influence disease severity. The mechanism(s) leading to altered gut homeostasis and barrier breakdown in inflammatory arthritis are poorly understood. In the present study, we found a significant reduction in intestinal concentrations of several proresolving mediators during inflammatory arthritis, including downregulation of the gut-protective mediator resolvin D5_n-3 DPA_ (RvD5_n-3 DPA_). This was linked with increased metabolism of RvD5_n-3 DPA_ to its inactive 17-oxo metabolite. We also found downregulation of IL-10 expression in the gut of arthritic mice that was coupled with a reduction in IL-10 and IL-10 receptor (IL-10R) in lamina propria macrophages. These changes were linked with a decrease in the number of mucus-producing goblet cells and tight junction molecule expression in the intestinal epithelium of arthritic mice when compared with naive mice. *P. gingivalis* inoculation further downregulated intestinal RvD5_n-3 DPA_ and *Il-10* levels and the expression of gut tight junction proteins. RvD5_n-3 DPA_, but not its metabolite 17-oxo-RvD5_n-3 DPA_, increased the expression of both IL-10 and IL-10R in macrophages via the upregulation of the aryl hydrocarbon receptor agonist l-kynurenine. Administration of RvD5_n-3 DPA_ to arthritic *P. gingivalis*–inoculated mice increased intestinal *Il-10* expression, restored gut barrier function, and reduced joint inflammation. Together, these findings uncover mechanisms in the pathogenesis of rheumatoid arthritis, where disruption of the gut RvD5_n-3 DPA_–IL-10 axis weakens the gut barrier, which becomes permissive to the pathogenic actions of the pathobiont *P. gingivalis*.

## Abstract 2

Loss of resolution mechanisms in the intestinal epithelium facilitate gut barrier breakdown by the pathobiont *Porphyromonas gingivalis*, leading to increased joint inflammation.

## Introduction

Rheumatoid arthritis (RA) is a chronic inflammatory disease primarily affecting the joints (1) and a leading cause of disability in Western societies (2). Environmental factors, such as mucosal barrier insults, are implicated in RA pathogenesis (3). These, together with genetic susceptibility that mainly affects immune-regulatory genes (4), drive the development of autoimmunity. Yet the mechanisms causing RA and influencing disease severity and flares remain elusive (5). Consequently, current therapeutic strategies in RA are primarily aimed at mitigating the symptoms rather than treating the cause. As a result, antirheumatic drugs need to be administered on a continuous basis to manage the symptoms, which leads to a number of unwanted side effects such as immunosuppression (6, 7) and inhibition of resolution and repair processes (8, 9).

The intestinal barrier is the site of the most intense host-microbiota interactions, and as such must be meticulously regulated to facilitate host-beneficial microbial activity, uptake of nutrients, and protection of commensals on its surface as well as from invasion by luminal microbes. In addition to the physical separation afforded by the inner mucus layer (10) and biochemical and immunological elements (11–13), intestinal paracellular permeability is regulated by cell-cell junction proteins of the gut epithelium (14). Observational studies suggest a link between RA, gut inflammation (15–17), and dysbiotic microbiota, with a number of bacteria that form part of the normal microbiota being implicated in propagating disease in RA (18). Among the bacteria proposed to be involved in disease propagation in arthritis is the pathobiont *Porphyromonas* *gingivalis,* which under homeostatic conditions forms part of the oral microbiota. *P*. *gingivalis* was recently found to promote gut barrier breakdown, leading to the systemic translocation of bacteria and increased disease severity (19). Of note, the underlying mechanisms facilitating barrier breach by this pathobiont during inflammatory arthritis remain of interest (18).

Lipid mediators (LMs) are central orchestrators of the initiation and resolution phases in acute, self-resolving inflammation (6). They include the arachidonic acid–derived prostaglandins (PGs), thromboxanes (TXs), and leukotrienes (LTs) (20, 21), which together with proinflammatory cytokines amplify the host response during early phases of acute inflammation. Studies assessing the mechanisms elicited by *P*. *gingivalis* in promoting inflammation demonstrate that this bacterium upregulates prostaglandin biosynthesis, leading to innate immune cell activation (22). Recent investigations into mechanisms regulating the termination of inflammation uncovered a temporally controlled LM class switch leading to the formation of potent mediators produced via the stereoselective conversion of essential fatty acids, including the omega-3 fatty acids docosahexanoic acid (DHA) and n-3 docosapentaenoic acid (DPA) (23), which actively drive the resolution of inflammation (24). These mediators are classified into 4 families that include the resolvins (Rvs), protectins (PDs), and maresins (MaRs), which are collectively termed specialized proresolving mediators (SPMs). SPMs reduce neutrophil–endothelial cell interactions and promote the recruitment of nonphlogistic monocytes and resolution-phase macrophages to the site of inflammation (6, 25). They promote tissue repair, and the clearance of apoptotic cells, bacteria, and cellular debris by macrophages (6). Their tissue homeostasis–promoting actions also play a critical role in the intestinal mucosa, where they regulate barrier function and tissue integrity (26, 27).

Given (i) the role that SPMs play in maintaining tissue homeostasis, including in the gut (24, 27), and (ii) that disruption of SPM pathways is proposed to be a central mechanism in the pathogenesis of many chronic diseases (14, 26, 28, 29), herein we questioned whether during RA, disruption of proresolving pathways in the gut facilitates the pathogenic behavior of the pathobiont *P*. *gingivalis*. Results from these experiments demonstrate that intestinal macrophages from arthritic mice display an altered phenotype and LM profile, with a downregulation in the expression of key protective molecules including IL-10R and RvD5_n-3 DPA_. This was linked with a reduction in the number of mucus-producing goblet cells and weakening of the gut barrier. In this environment, inoculation with *P*. *gingivalis* led to bacterial translocation, dysregulation of lamina propria immune responses, and exacerbated joint inflammation. Thus, these findings indicate that in inflammatory arthritis, altered mucosal barrier function predisposes the host to pathogenic actions of pathobionts that in turn exacerbate joint inflammation by promoting the translocation of intestinal bacteria.

## Results

### Arthritis dysregulates the gut barrier function.

**Figure 1:**
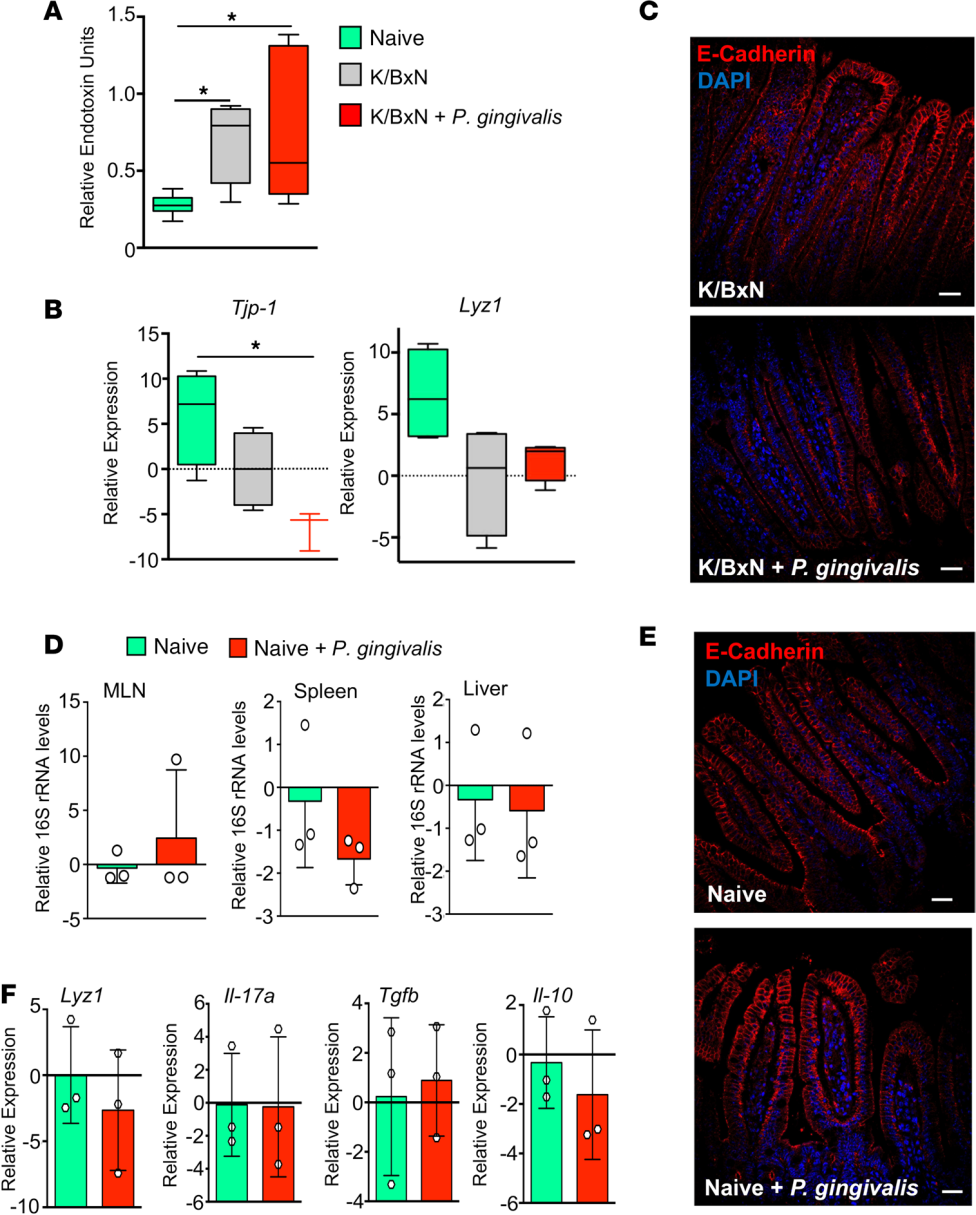
Inoculation of arthritic but not naive mice with *P. gingivalis* promotes gut barrier breakdown. (**A**–**C**) Mice were inoculated with *P*. *gingivalis* (10^9^ CFU per mouse) or given vehicle (PBS) on days –1, 1, and 3 and injected with K/BxN serum (50 μL per mouse, i.p.; days 0 and 2). Tissues were harvested on day 8 after K/BxN administration, and (**A**) plasma endotoxin concentrations were determined. (**B**) Gene expression of tight junction protein *Tjp1* and antimicrobial *Lyz1*. Results for **A** and **B** are mean ± SEM. *n* = 3–4 mice per group from 2 independent experiments. *P < 0.05 using Kruskall-Wallis test followed by Dunn’s post hoc test. (**C**) Representative images of E-cadherin staining in arthritic mice (K/BxN) inoculated with or without *P*. *gingivalis* (scale bars: 25 μm). Results are presented as mean ± SEM. *n* = 4 mice per group from 2 independent experiments. (**D**) Mice were gavaged with *P*. *gingivalis* (10^9^ CFU, days –1, 1, 3) or vehicle. 16S rRNA gene levels were measured by 16S qPCR in mesenteric lymph nodes (MLNs), spleens, and livers of naive control mice or *P*. *gingivalis*–inoculated mice (Naive + *P*. *gingivalis*) to assess breach of bacteria across the gut barrier. Results are mean ± SEM for *n* = 3 mice per group; unpaired *t* test with Welch’s correction. (**E**) Representative images of E-cadherin (red) and DAPI staining (blue) of ileal tissue from naive mice or *P*. *gingivalis*–inoculated mice (Naive + *P*. *gingivalis*). Representative of *n* = 4 mice from 2 independent experiments. Scale bars: 25 μm. (**F**) Gene expression levels of intestinal epithelium–secreted antimicrobial *Lyz1* and cytokines *Il17a, Tgfb*, and *Il10* in intestines of naive and nonarthritic *P*. *gingivalis*–inoculated (Naive + *P*. *gingivalis*) mice. Results are mean ± SEM for *n* = 3 mice per group.

To gain insights into mechanism(s) leading to increased disease severity by *P*. *gingivalis* in RA, we first investigated whether barrier function was altered in inflammatory arthritis. In mice in which inflammatory arthritis had been induced by K/BxN serum injection, we found a marked increase in plasma endotoxin concentrations when compared with naive mice (Figure 1A), in accordance with findings made in patients with arthritis (16). These increases were linked with a downregulation of the tight junction molecule tight junction protein-1 (*Tjp1*, encoding zona occludens-1, ZO-1) (30, 31) and lysozyme 1 (*Lyz1*), which encodes for a major antimicrobial protein expressed constitutively in the small intestine (11) (Figure 1B). In order to test whether the ability of *P*. *gingivalis* to aggravate joint inflammation was shared with other bacterial species, we next inoculated arthritic mice with the commensal microbe *Bacteroides thetaiotaomicron* and assessed disease severity. Here we found that in contrast to *P*. *gingivalis, B*. *thetaiotaomicron* inoculation did not alter disease kinetics or severity (Supplemental Figure 1A; supplemental material available online with this article; https://doi.org/10.1172/jci.insight.125191DS1). These results demonstrate that the observed regulation of disease severity by *P*. *gingivalis* was not a pan-microbial effect. In order to gain better insight into the mechanisms by which *P*. *gingivalis* regulated disease severity in inflammatory arthritis, we next assessed whether the actions of *P*. *gingivalis* in promoting gut barrier breakdown were limited to arthritic mice or whether this also occurred in healthy mice. In accordance with published findings (32), inoculation of arthritic mice with *P*. *gingivalis* led to a breakdown in the gut barrier, with a significant increase in overall bacterial load in the colonic inner mucus layer and the lamina propria, normally impenetrable to bacteria (10); an increase in bacterial 16S ribosomal DNA (rDNA) levels in lymphoid tissues (Supplemental Figure 1, B and C); and an increase in plasma endotoxin concentrations in *P*. *gingivalis*–inoculated mice (Figure 1A). It also led to a significant reduction in the expression of several tight junction molecules, including *Tjp1* and E-cadherin (Figure 1, B and C). Of note, such a breach of the mucosal barrier was not observed in nonarthritic mice inoculated with *P*. *gingivalis* (Figure 1, D–F). Thus, these findings suggest that inflammatory arthritis results in changes in gut barrier function that facilitate barrier breach by gut bacteria following *P*. *gingivalis* inoculation.

### Intestinal resolution pathways are dysregulated during inflammatory arthritis.

**Figure 2:**
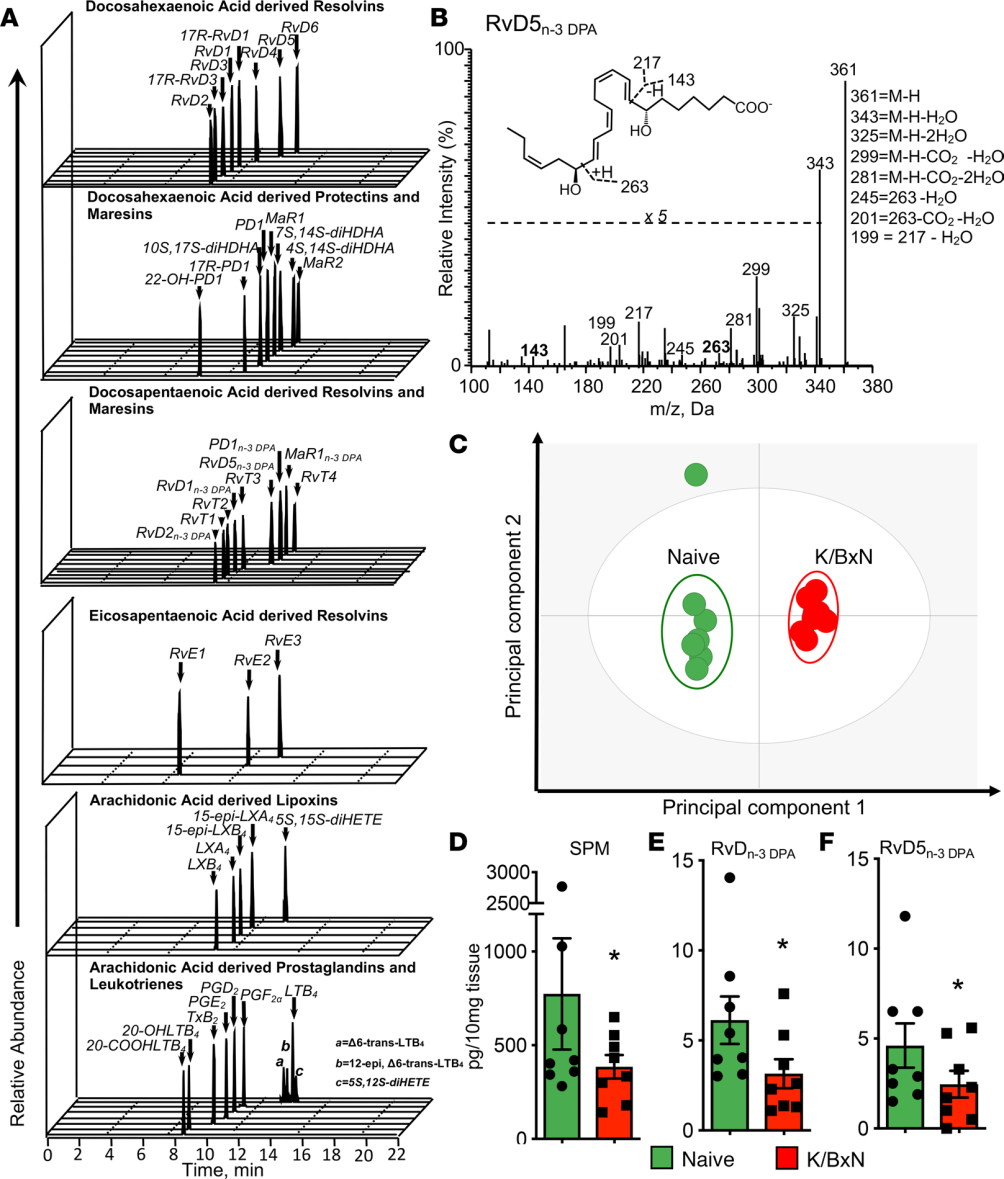
Arthritis dysregulates intestinal lipid mediator profiles. Arthritis was initiated by injection of K/BxN serum (50 μL per mouse, i.p.; days 0 and 2). On day 8 ilea were harvested from arthritic and naive mice and lipid mediators identified and quantified using lipid mediator profiling (see Methods for details). (**A**) Representative multiple reaction monitoring (MRM) traces for identified lipid mediators. (**B**) Representative MS/MS spectrum employed for the identification of RvD5_n-3 DPA_; inset, diagnostic ions; M, molecular mass. (**C**) Orthogonal partial least squares discriminant analysis (oPLS-DA) of intestinal lipid mediator profiles. Cumulative tissue concentrations for SPMs (i.e., arachidonic-, eicosapentaenoic acid–, n-3 docosapentaenoic– [DPA–], and docosahexaenoic acid–derived [DHA-derived] proresolving mediators) (**D**), RvD_n-3 DPA_ (i.e., RvD1_n-3 DPA_, RvD2_n-3 DPA_, and RvD5_n-3 DPA_) (**E**), and RvD5_n-3 DPA_ (**F**). Results for **A** and **B** are representative of *n* = 24 mice; for **C** are representative of *n* = 8 mice per group; for **D**–**F** are mean ± SEM for *n* = 8 mice per group from 2 independent experiments; *P ≤ 0.05 versus naive using Mann-Whitney *U* test. Results are expressed as pg/10 mg tissue.

Resolution mechanisms are important in maintaining tissue homeostasis and function, and recent studies demonstrate that dysregulation of proresolving pathways in the gut is linked with intestinal inflammation (27). In order to investigate whether during inflammatory arthritis gut resolution responses are altered and thus may increase susceptibility to *P*. *gingivalis*, we assessed intestinal LM concentrations during inflammatory arthritis. Using liquid chromatography– tandem mass spectrometry–based (LC-MS/MS–based) LM profiling, we identified mediators from all 4 major fatty acid bioactive metabolomes, including lipoxygenase- and cyclooxygenase-derived LMs that were identified in accordance with published criteria (33) (Figure 2, A and B). Multivariate analysis of identified mediators gave 2 distinct clusters, demonstrating a marked shift in intestinal LM concentrations in arthritic mice (Figure 2C). This shift was linked with a reduction in proresolving mediator concentrations in arthritic mice. Among the mediator families that were downregulated, we measured a significant reduction in the recently uncovered n-3 DPA–derived resolvins (RvD_n-3 DPA_), including the gut-protective RvD5_n-3 DPA_ (27) (Figure 2, D–F, and Supplemental Table 1). Together, these findings suggest that resolution mechanisms are disrupted in the arthritic gut.

### Altered intestinal epithelial function and macrophage phenotype during inflammatory arthritis.

**Figure 3:**
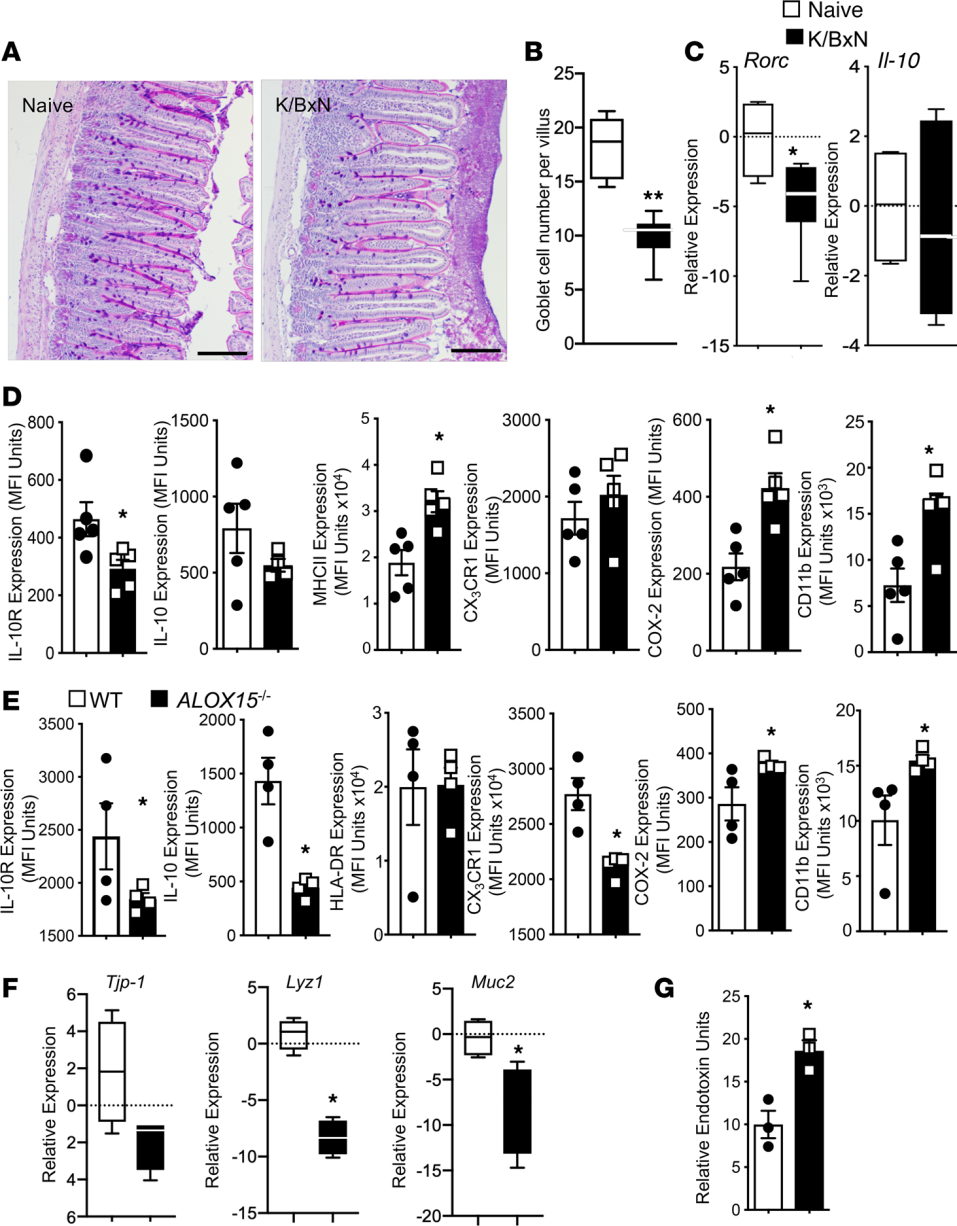
Inflammatory arthritis alters the expression of intestinal epithelial barrier components and lamina propria macrophage phenotype: a role for SPM in the maintenance of gut barrier function. Arthritis was initiated by injection of K/BxN serum (50 μL per mouse, i.p.; days 0 and 2). On day 8 ilea were harvested from arthritic and naive mice and mucus-producing goblet cells were (**A**) stained using periodic acid–Schiff (PAS) stain and (**B**) enumerated. Scale bars: 100 μm. (**C**) The expression of *Rorc* and *Il-10* in the small intestine was determined using qRT-PCR. (**D**) Lamina propria leukocytes were isolated, and the expression of the indicated lineage markers determined using flow cytometry. Results for **A** are representative of *n* = 4 mice per group from 2 independent experiments. Results for **B**–**D** are mean ± SEM. *n* = 4–5 mice per group from two independent experiments. *P ≤ 0.05, **P ≤ 0.01, versus naive mice using Mann-Whitney *U* test. (**E**) Lamina propria macrophages were isolated from naive WT and *Alox15^–/–^* mice, and the expression of lineage markers was determined using flow cytometry. (**F**) Tissues were collected from naive WT and *Alox15^–/–^* mice, and gene expression was assessed using qRT-PCR. (**G**) Endotoxin levels were measured in plasma. Results for **E**–**G** are mean ± SEM. *n* = 4–5 mice per group from 2 independent experiments. *P < 0.05 versus WT mice using Mann-Whitney *U* test.

Having found a shift in mediator profiles with a dysregulation in several tissue- and gut-protective mediators during arthritis, we next questioned whether this alteration was also linked with changes in intestinal barrier function. In the intestines of arthritic mice, together with a reduction in genes involved in epithelial barrier function, including *Tjp1* and *Lyz1* (Figure 1)*,* we found a significant reduction in the number of mucus-producing goblet cells (Figure 3, A and B) and a downregulation of the immune-regulating genes *Rorc* and *Il-10* (Figure 3C).

Recent studies highlight a central role for the macrophage IL-10 pathway in maintaining gut barrier function (34, 35). Thus, we investigated expression of IL-10 and IL-10R in lamina propria macrophages together with expression of macrophage phenotypic markers. Flow cytometric analysis demonstrated significant downregulation of both of these molecules and upregulation of the activation markers CD11b and MHCII in arthritic mice (Figure 3D). These results suggest that in inflammatory arthritis there was a shift in gut macrophage phenotype, with a downregulation of the IL-10 pathway, that was associated with an alteration of gut barrier function.

### Loss of SPM production in the lamina propria alters gut barrier function.

Having found that SPMs are dysregulated in the lamina propria of arthritic mice, we questioned whether this loss was linked with an alteration in macrophage IL-10 axis (i.e., IL-10 and IL-10R) and barrier function. For this purpose we next assessed the expression of IL-10 and IL-10R in lamina propria macrophages from mice deficient in 15-lipoxygenase (ALOX15), the initiating enzyme in the resolvin, protectin, and lipoxin biosynthetic pathway (23). Flow cytometric analysis of lamina propria macrophages demonstrated a significant downregulation in expression of both IL-10 and IL-10R, as well as an upregulation of the activation markers COX-2 and CD11b in macrophages from *Alox15*-deficient mice when compared with lamina propria macrophages from WT mice (Figure 3E). In the intestines of *Alox15*-deficient mice, we also found a significant downregulation in expression of genes involved in epithelial barrier function, including *Tjp1* and *Muc2* (Figure 3F), as well as higher plasma endotoxin concentrations (Figure 3G). Thus, these results support a role for intestinal SPM production in regulating macrophage phenotype and barrier function.

### RvD5_n-3 DPA_ rectifies LM profiles of intestinal macrophages following arthritogenic challenge.

**Figure 4:**
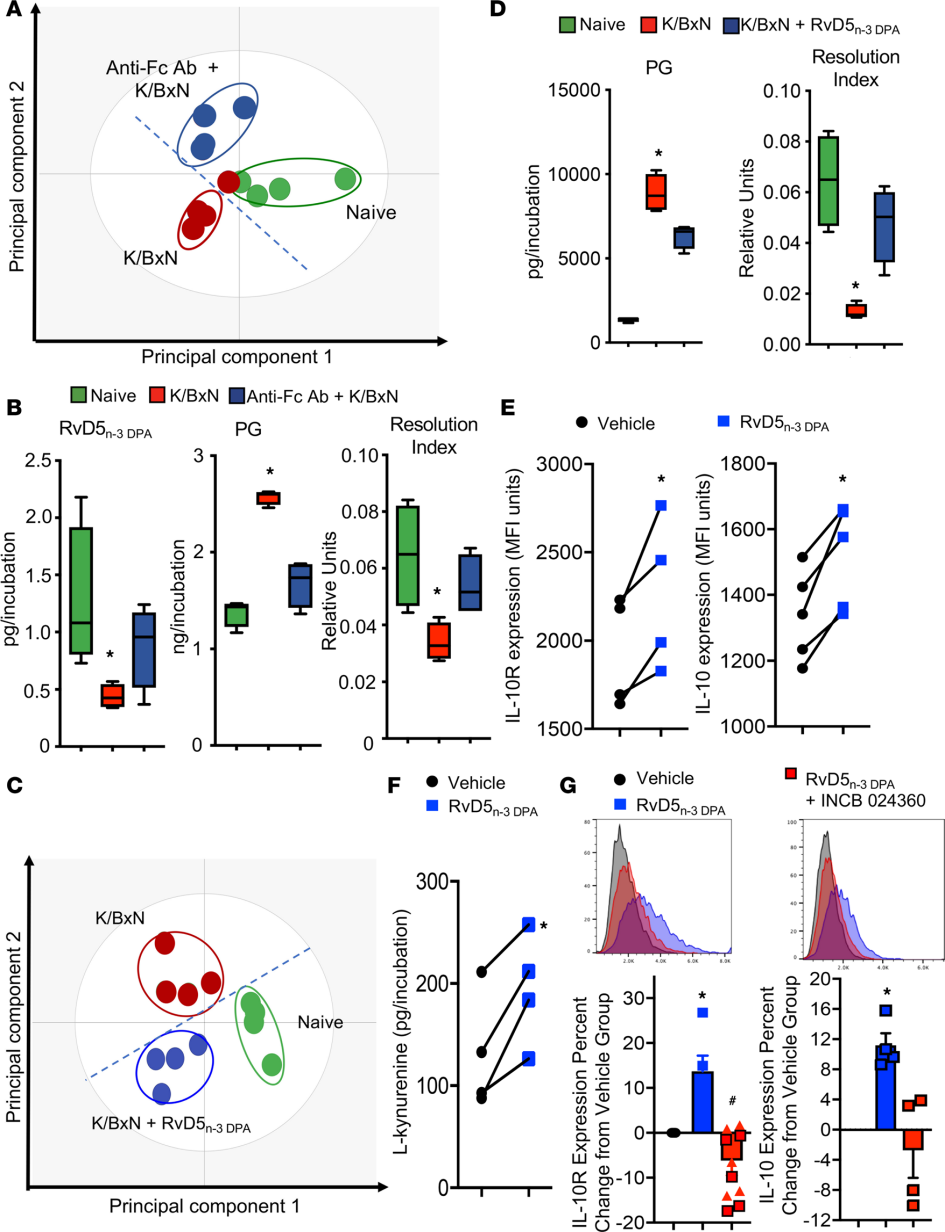
RvD5_n-3 DPA_ rectifies intestinal macrophage lipid mediator profiles and upregulates macrophage IL-10 and IL-10R expression via the activation of IDO and AHR. (**A** and **B**) Lamina propria macrophages were isolated from naive mice and incubated with vehicle (Naive), isotype control antibodies (K/BxN), or anti-CD16 and anti-CD32 antibodies (anti-Fc Ab + K/BxN) for 20 minutes (37°C). Cells were then incubated with vehicle or K/BxN serum (1:1000 dilution; 16 hours at 37°C). Incubations were quenched with ice-cold methanol and lipid mediators identified and quantified using lipid mediator profiling. (**A**) oPLS-DA of lipid mediator profiles. (**B**) Concentrations of RvD5_n-3 DPA_ (left panel), prostaglandins (PG; center), and ratio of proresolving mediators to proinflammatory eicosanoids (Resolution Index; right). Results are mean ± SEM, with horizontal bars depicting mean values. *n* = 4 mice per group. *P < 0.05 versus naive using Kruskall-Wallis test followed by Dunn’s post hoc test. (**C** and **D**) Lamina propria macrophages were isolated from naive mice and arthritic mice and then incubated with either vehicle (K/BxN) or RvD5_n-3 DPA_ (10 nM; K/BxN + RvD5_n-3 DPA_) for 20 minutes (37°C; 16 hours). Incubations were quenched with ice-cold methanol, and lipid mediators were identified and quantified using lipid mediator profiling. (**C**) oPLS-DA of lipid mediator profiles. (**D**) Concentrations of prostaglandins (left panel) and resolution index (right). Results are mean ± SEM. *n* = 4 mice per group. *P < 0.05 versus naive using Kruskall-Wallis test followed by Dunn’s post hoc test. (**E**) Bone marrow–derived macrophages were incubated with 10 nM RvD5_n-3 DPA_ or vehicle (16 hours, 37°C), and IL-10R and IL-10 expression was determined using flow cytometry. (**F**) Bone marrow–derived macrophages were incubated with 10 nM RvD5_n-3 DPA_ or vehicle (2 hours, 37°C) and concentrations of l-kynurenine measured by LC-MS/MS. (**G**) Bone marrow–derived macrophages were incubated with 20 μM INCB 024360 or vehicle (30 minutes, 37°C), then with 10 nM RvD5_n-3 DPA_ or vehicle (16 hours, 37°C) and IL-10R and IL-10 expression determined using flow cytometry (lower panels). Representative histograms are shown (upper panels). Results for **E**–**G** are mean ± SEM. *n* = 4–5 mice per group from 2 independent experiments. *P < 0.05 versus vehicle group using Mann-Whitney *U* test for **E** and **F**, and Kruskall-Wallis test followed by Dunn’s post hoc test for **G**.

Given the central role that macrophages play in maintaining gut barrier function (35), we next studied the mechanisms leading to an altered lamina propria macrophage phenotype in inflammatory arthritis. For this purpose we incubated macrophages with arthritogenic serum that is rich in immune complexes, factors that are central to disease onset/propagation in arthritis (36, 37). Incubation of lamina propria macrophages with arthritogenic serum led to a significant shift in the macrophage biosynthetic profile, with a marked downregulation in RvD5_n-3 DPA_ and an upregulation of proinflammatory eicosanoids. This was linked with an overall inflammatory LM profile as measured by a reduction in the resolution index (i.e., the sum of proresolving mediators divided by the sum of proinflammatory eicosanoids) (38) (Figure 4, A and B). Of note, incubation of these macrophages with blocking antibodies against Fc receptors in part reversed the shift in macrophage LM profiles (Figure 4, A and B, and Supplemental Table 2). These findings suggest that during inflammatory arthritis, immune complexes may disrupt proresolving mediator pathways in lamina propria macrophages, dysregulating the production of tissue-protective SPMs.

Having found a significant reduction in the concentrations of the gut-protective RvD5_n-3 DPA_ in vivo and with lamina propria macrophages in vitro, we next investigated whether this mediator was involved in regulating macrophage responses during inflammatory arthritis. Here we assessed whether RvD5_n-3 DPA_ rectified LM profiles of lamina propria macrophages from arthritic mice. Using partial least squares discriminant analysis (PLS-DA), we found that the LM profile cluster of macrophages from arthritic mice incubated with RvD5_n-3 DPA_ was shifted away from the profile cluster of macrophages from arthritic mice not incubated with the SPMs and toward the LM cluster from naive mice (Figure 4C). This shift was associated with a decrease in the concentration of inflammatory prostaglandins, an increase in the proresolving LM concentrations, and a restoration of the resolution index (Figure 4D and Supplemental Table 3). Together, these results suggest that RvD5_n-3 DPA_ regulates lamina propria macrophage responses and that in inflammatory arthritis, immune complexes disrupt the production of this protective mediator.

### RvD5_n-3 DPA_ upregulates IL-10R and IL-10 expression via activation of the aryl hydrocarbon receptor.

Loss of expression of *Il-10* in the lamina propria and of its receptor in lamina propria macrophages leads to a disruption of gut barrier function and to spontaneous gut inflammation in mice lacking these proteins (34, 39). Since we found an alteration in expression of both IL-10 and IL-10R in intestinal macrophages from arthritic mice that was linked with a reduction in RvD5_n-3 DPA_, we next queried whether this SPM regulated IL-10 and IL-10R in macrophages. Flow cytometric analysis of bone marrow–derived macrophages indicated that incubation with RvD5_n-3 DPA_ upregulated expression of both IL-10 and IL-10R in these cells (Figure 4E).

Recent studies demonstrate that the promoter region of IL-10R contains aryl hydrocarbon receptor (AHR) response elements and that activation of this receptor by the tryptophan metabolite l-kynurenine upregulates IL-10R expression (40). Therefore, we questioned whether activation of this pathway also mediated the upregulation of IL-10R by RvD5_n-3 DPA_. We first assessed the expression of l-kynurenine in cells incubated with or without RvD5_n-3 DPA_, finding that this SPM significantly upregulated l-kynurenine expression (Figure 4F). Furthermore, incubation of bone marrow–derived macrophages with INCB 024360-analog — an inhibitor of indoleamine 2,3-dioxygenase (IDO), the enzyme that converts tryptophan to l-kynurenine — inhibited the upregulation of IL-10R by RvD5_n-3 DPA_ (Figure 4G). Of note, in these incubations we found that inhibition of IDO also decreased IL-10 expression (Figure 4G).

### Immune complexes promote the inactivation of RvD5_n-3 DPA_ via upregulating 15-PGDH.

To establish the mechanisms leading to altered barrier function during inflammatory arthritis, we next investigated the processes leading to downregulation of RvD5_n-3 DPA_ in the intestinal epithelium during inflammatory arthritis. We first investigated the activity of ALOX5 and ALOX15, the RvD5_n-3 DPA_ biosynthetic enzymes (23), in intestinal tissues and lamina propria macrophages. Using LM profiling, we assessed the concentrations of 7-HDPA and 17-HDPA, markers of ALOX5 and ALOX15 activity, respectively. In small intestines from arthritic mice, we found that concentrations of 17-HDPA were increased, whereas those of 7-HDPA were essentially identical to those in naive mice (Supplemental Figure 2A). In lamina propria macrophage incubations, we found that addition of arthritogenic serum upregulated expression of both molecules (Supplemental Figure 2B). Thus, these results suggest that during arthritic inflammation, the activity of ALOX5 and ALOX15 is not reduced.

**Figure 5:**
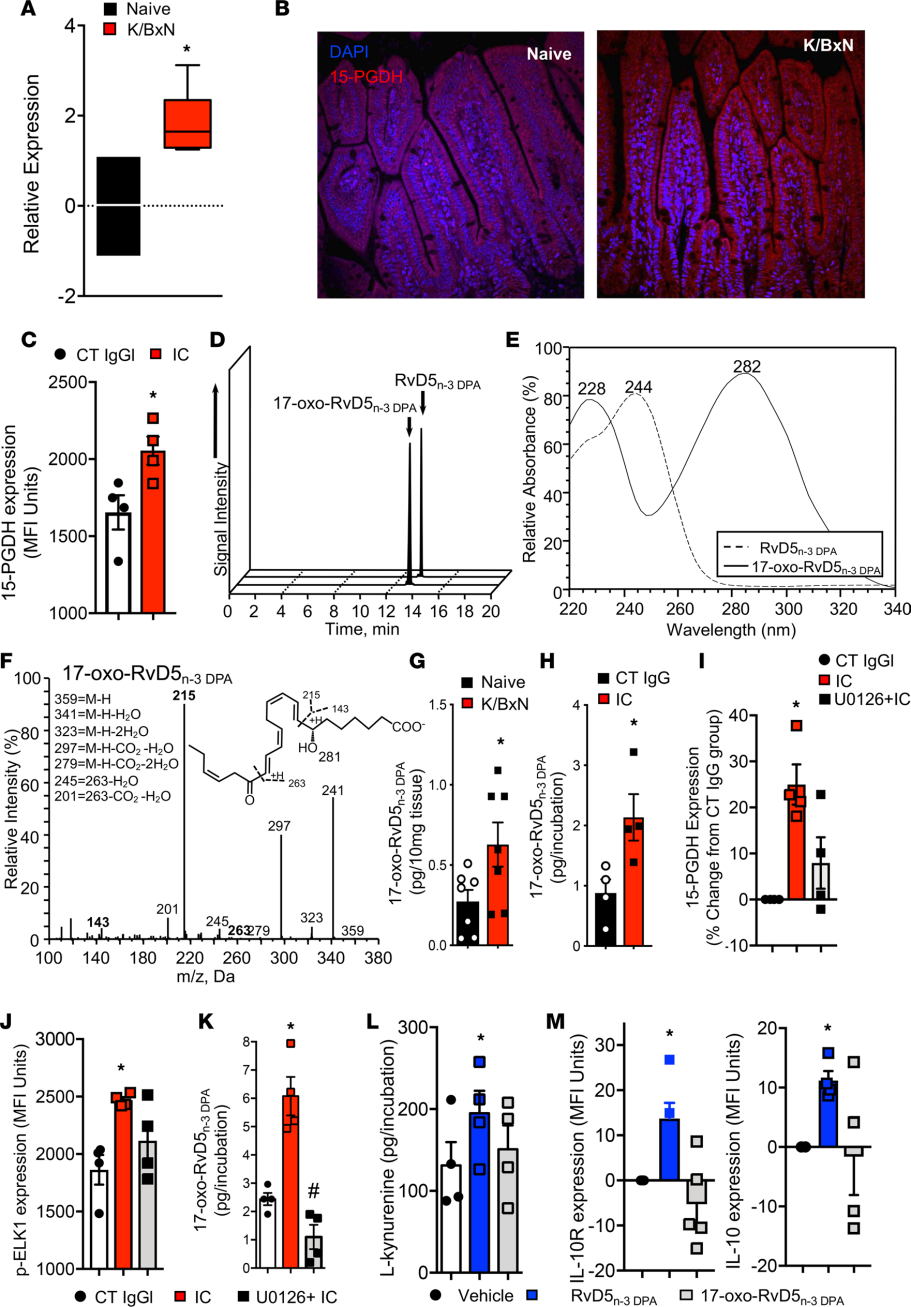
Upregulation of 15-PGDH in the lamina propria during inflammatory arthritis promotes the inactivation of RvD5_n-3 DPA_. Inflammatory arthritis was initiated as detailed in Figure 1, and small intestines were collected on day 8 after initiation. (**A**) mRNA expression of 15-PGDH. (**B**) Immunofluorescence analysis of 15-PGDH expression in the small intestine. Original magnification, ×60. (**C**) Bone marrow macrophages were incubated with immune complexes (IC) or control IgG (16 hours, 37°C), and the expression of 15-PGDH was assessed using flow cytometry. Results for **A** and **C** are mean ± SEM. For **A**, *n* = 7 mice per group; for **C**, *n* = 4 mice per group from 2 independent experiments. Results for **B** are representative of *n* = 4 mice per group from 2 independent experiments. (**D**–**F**) RvD5_n-3 DPA_ was incubated with hr15-PGDH (0.5 U, room temperature, 45 minutes). Products were extracted and lipid mediators were identified using liquid chromatography tandem mass spectrometry and reversed-phase UV-HPLC. (**D**) MRM chromatograms of 361 > 199 (RvD5_n-3 DPA_) and 359 > 215 (17-oxo-RvD5_n-3 DPA_). (**E**) Online UV chromatogram for RvD5_n-3 DPA_ and 17-oxo-RvD5_n-3 DPA_. (**F**) MS/MS spectrum employed in the identification of 17-oxo-RvD5_n-3 DPA_. Results are representative of *n* = 4 incubations. (**G**) Arthritis was initiated as in Figure 1, small intestines harvested on day 8 after initiation, products were extracted, and the concentration of 17-oxo-RvD5_n-3 DPA_ was determined using LC-MS/MS. (**H**) Bone marrow–derived macrophages were incubated with vehicle or immune complexes (37°C, 16 hours) and 17-oxo-RvD5_n-3 DPA_ concentrations were determined using LC-MS/MS. Results are mean ± SEM *n* = 4–5 mice per group from 2 independent experiments. (**I**–**K**) Bone marrow–derived macrophages were incubated with vehicle or U0126 (20 μM, 37°C, 1 hour), then with either vehicle or immune complexes (37°C, 16 hours), and the expression of (**I**) 15-PGDH and (**J**) ELK1 and was investigated using flow cytometry. (**K**) Concentrations of 17-oxo-RvD5_n-3 DPA_ were determined using lipid mediator profiling. (**L**) Bone marrow–derived macrophages were incubated with RvD5_n-3 DPA_ (10 nM), 17-oxo-RvD5_n-3 DPA_ (10 nM), or vehicle (37°C, 2 hours), and concentrations of l-kynurenine determined using LC-MS/MS. (**M**) Cells were incubated as in **K** for 16 hours (37°C), and expression of IL-10R and IL-10 was assessed using flow cytometry. Results for **I**–**M** are mean ± SEM. *n* = 4 mice per group from 2 independent experiments. *P < 0.05 using Kruskall-Wallis test followed by Dunn’s post hoc test.

Another point of regulation for tissue SPM concentration is their further metabolism, which may also lead to the inactivation of these protective mediators (41). Thus, we next questioned whether the reduction in RvD5_n-3 DPA_ during inflammatory arthritis was due to increased local metabolism of this SPM. One of the enzymes responsible for the inactivation of SPMs is 15-prostaglandin dehydrogenase (15-PGDH) (41). This enzyme oxidizes secondary alcohols to ketones, leading, in the resulting product, to the loss in protective actions of the parent SPM (41). Therefore, we first assessed whether 15-PGDH was upregulated in the intestines of arthritic mice. Using quantitative real-time PCR (qRT-PCR) and immunohistochemistry, we found increased expression of both mRNA and protein of this enzyme in small intestines from diseased mice compared with naive mice (Figure 5, A and B). Because during inflammatory arthritis the production of RvD5_n-3 DPA_ was altered in lamina propria macrophages and these cells are activated by immune complexes via the Fc receptors, we next assessed whether immune complexes upregulate the expression of 15-PGDH in macrophages. For this purpose, we incubated bone marrow–derived macrophages with isolated immune complexes or control IgG and assessed 15-PGDH expression by flow cytometry, finding that indeed immune complexes significantly upregulated the expression of 15-PGDH in these cells (Figure 5C).

Having found that this enzyme was upregulated, we next assessed whether it was linked with increased metabolism of RvD5_n-3 DPA_. For this purpose, we first established the identity of the major products for the enzyme when RvD5_n-3 DPA_ was the substrate. In incubations of RvD5_n-3 DPA_ with human recombinant 15-PGDH, we identified one major product in reversed-phase UV HPLC that carried a UV chromophore with a λ_max_^MeOH^ of 282 nm with a shoulder at 228 nm. In LC-MS/MS this product gave a peak with retention time of 13.7 minutes, and assessment of ions in the MS/MS spectrum demonstrated that it carried a ketone at carbon position 17, indicating it was 17-oxo-RvD5_n-3 DPA_ (Figure 5, D–F). Having established the identity of this product, we next investigated whether it was present in intestines from arthritic mice and whether the concentrations of 17-oxo-RvD5_n-3 DPA_ were increased during inflammatory arthritis. Quantitation of this molecule using multiple reaction monitoring demonstrated an increase in 17-oxo-RvD5_n-3 DPA_ concentrations in arthritic intestines (Figure 5G). Of note, this product was also elevated in incubations of bone marrow–derived macrophages with immune complexes (Figure 5H).

Recent studies demonstrate that the expression of 15-PGDH is under the control of the ETS transcription factor (ELK1), which when phosphorylated by ERK1 and ERK2 translocates to the nucleus and upregulates expression of the enzyme (42). Given that Fc receptor activation regulates ERK activity, we next assessed whether immune complexes, which are central aspect of RA pathogenesis (36, 37), from arthritic serum upregulate 15-PGDH expression in macrophages via the ERK1/2-ELK1 pathway. Incubation of bone marrow–derived macrophages with immune complexes increased 15-PGDH expression (Figure 5I), an increase that was associated with an upregulation in ELK1 phosphorylation when compared with cells incubated with control antibodies (Figure 5J). Of note preincubation of macrophages with the ERK1/2 inhibitor U0126 reduced ELK1 phosphorylation and reversed the immune complex–mediated upregulation of 15-PGDH expression. In these incubations we also found a reduction in the concentrations of 17-oxo-RvD5_n-3 DPA_ when compared with cells incubated with vehicle alone (Figure 5, G, H, and K).

Having established that immune complexes upregulate 15-PGDH expression and this leads to an increase in the further metabolism of RvD5_n-3 DPA_, we next tested whether this pathway was indeed responsible for downregulating the expression of IL-10 and its receptor on macrophages. For this purpose, we incubated bone marrow–derived macrophages with either RvD5_n-3 DPA_ or 17-oxo-RvD5_n-3 DPA_ and assessed the expression of these 2 proteins using flow cytometry. Here we found that the 17-oxo-RvD5_n-3 DPA_ did not increase the concentrations of l-kynurenine (Figure 5L) and failed to significantly upregulate the expression of IL-10R and IL-10 in bone marrow–derived macrophages (Figure 5M). Hence, these results support the hypothesis that further metabolism of RvD5_n-3 DPA_ in the arthritic gut contributes to the downregulation of the IL-10 axis.

### RvD5_n-3 DPA_ restores gut barrier function and immune responses in P.

**Figure 6:**
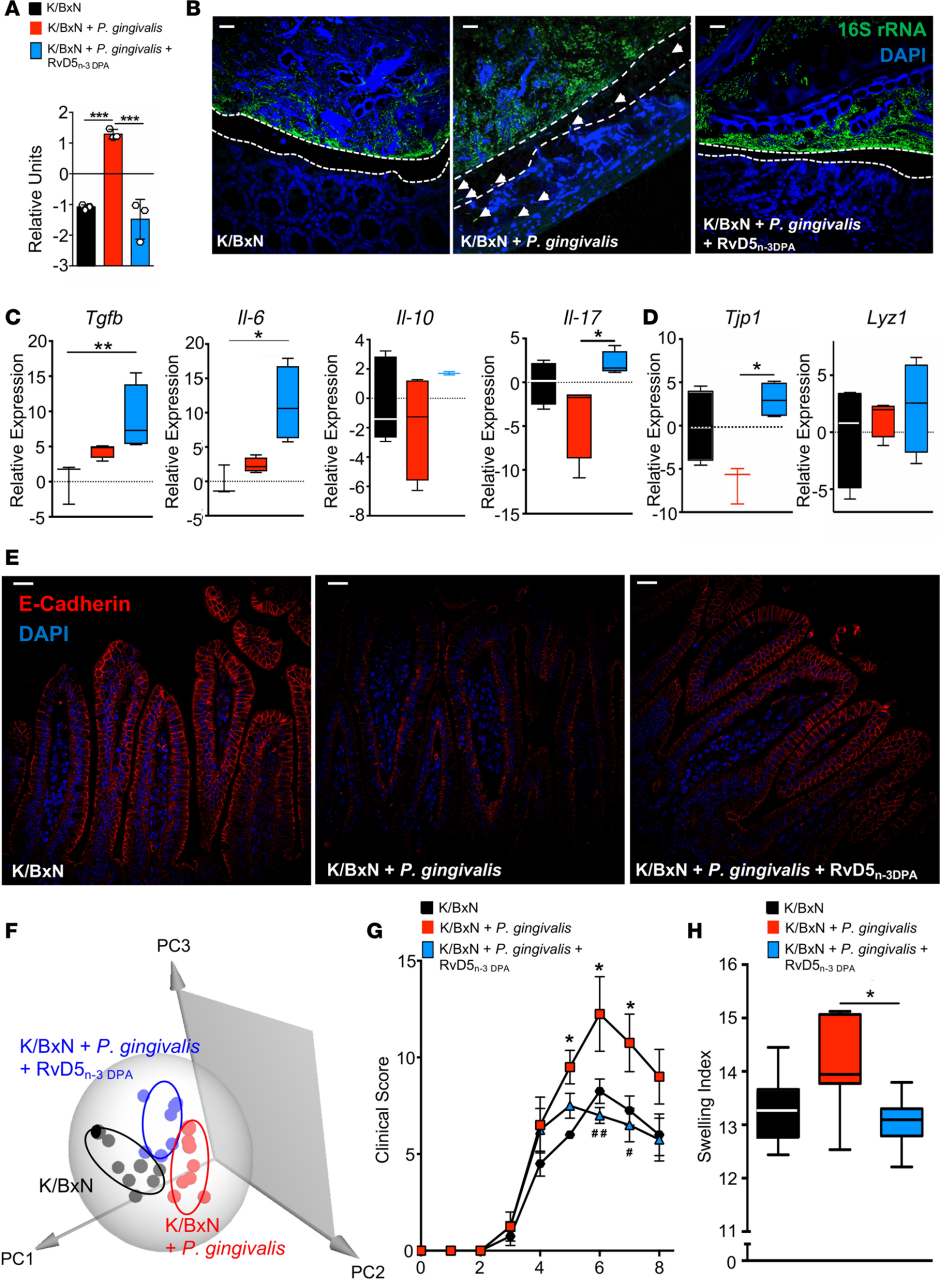
RvD5_n-3 DPA_ upregulates gut Il-10 expression and restores altered gut barrier function, reducing joint inflammation in *P. gingivalis*–inoculated arthritic mice. Mice were injected with K/BxN serum (50 μL per mouse, i.p.; days 0 and 2) and inoculated with *P*. *gingivalis* (10^9^ CFU per mouse) or given vehicle (PBS) on days –1, 1, and 3. In addition, mice were injected i.p. on days 3 and 5 with 200 ng per mouse of RvD5_n-3 DPA_ (K/BxN + *P*. *gingivalis* + RvD5_n-3 DPA_) or vehicle (saline plus 0.1% ethanol; K/BxN + *P*. *gingivalis*). Tissues were harvested on day 8. (**A**) 16S rRNA gene levels were measured by 16S qPCR in mesenteric lymph nodes (MLNs) to assess bacterial translocation across the gut barrier. Results are mean ± SEM and representative of *n* = 4 mice per group from 2 independent experiments; ***P ≤ 0.001 using Kruskall-Wallis test followed by Dunn’s post hoc test. (**B**) Representative images of 16S FISH using an Alexa Fluor 488–labeled 16S rRNA gene probe (green) to visualize bacteria and DAPI (blue) to visualize host cells in colons of arthritic vehicle-gavaged control mice and *P*. *gingivalis*–inoculated mice injected with vehicle or RvD5_n-3 DPA_. Dotted lines outline mucus layer; arrows denote bacterial invasion into the gut epithelium (scale bars: 25 μm). Results are representative of *n* = 4 mice per group from 2 independent experiments. Gene expression of (**C**) cytokines *Tgfb,* *Il6*, *Il10,* and *Il17a* , (**D**) the tight junction protein *Tjp1*, and the antimicrobial *Lyz1* was assessed in ileal mucosal tissue from arthritic mice gavaged with vehicle, inoculated with *P*. *gingivalis*, and injected i.p. with RvD5_n-3 DPA_ or controls. Results for **C** and **D** are mean ± SEM for *n* = 3–4 mice per group from 2 independent experiments; Kruskall-Wallis followed by Dunn’s post hoc test; *P ≤ 0.05, **P ≤ 0.01. (**E**) Immunofluorescence analysis of E-cadherin in small intestines from arthritic mice; scale bars: 25 μm. Results are representative of *n* = 4 mice per group from 2 independent experiments. (**F**) Ilea were harvested on day 8, and lipid mediators were identified and quantified using lipid mediator profiling in 7–8 mice per group from 2 independent experiments. (**G** and **H**) Mice were inoculated, challenged, and treated as above. (**G**) Clinical arthritis scores were recorded over time. Results are mean ± SEM for *n* = 4 mice/time point/group from 2 independent experiments; 2-way ANOVA followed by Bonferroni’s post hoc test; *P ≤ 0.05, K/BxN-injected, *P*. *gingivalis*–inoculated, vehicle-injected group (K/BxN + *P*. *gingivalis*) versus K/BxN-injected, vehicle-gavaged, vehicle-injected control group (K/BxN); ^#^*P* ≤ 0.05, ^##^*P* ≤ 0.01, K/BxN-injected, *P*. *gingivalis*–inoculated, RvD5_n-3 DPA_–injected group (K/BxN + *P*. *gingivalis* + RvD5_n-3 DPA_) versus K/BxN + *P*. *gingivalis*. (**H**) Swelling indices of edema formation on day 8 in paws and ankles of 8 mice per group from 2 independent experiments; *P ≤0.05 using Kruskall-Wallis followed by Dunn’s post hoc test.

*gingivalis–inoculated mice, reducing joint inflammation*. Having found that RvD5_n-3 DPA_ regulates the gut homeostatic IL-10 axis in macrophages in vitro, we next questioned whether administration of RvD5_n-3 DPA_ during inflammatory arthritis would upregulate IL-10 expression and rectify barrier function, protecting against *P*. *gingivalis*–mediated barrier breach. For this purpose, arthritis was initiated and mice were gavaged with *P*. *gingivalis* or vehicle, as above. Mice were then administered RvD5_n-3 DPA_ (200 ng in PBS, i.p. injection) or vehicle (0.1% ethanol in PBS) on days 3 and 5, and effects on the gut barrier were assessed. Quantitative PCR for 16S rDNA (16S qPCR) demonstrated a significant reduction in bacterial levels in mesenteric lymph nodes (MLNs) in mice given the SPM, as compared with mice given *P*. *gingivalis* alone (Figure 6A). In line with the rescue of barrier function, 16S fluorescence in situ hybridization (FISH) imaging demonstrated that the colon inner mucus layer and lamina propria of RvD5_n-3 DPA_–treated mice were free of bacteria (Figure 6B). Next, IL-10 expression was investigated in the intestinal mucosa. Administration of RvD5_n-3 DPA_ to *P*. *gingivalis*–inoculated arthritic mice upregulated *Il-10* expression when compared with mice given vehicle alone (Figure 6C). Of note, the expression of this intestinal homeostatic molecule was also higher than that observed in arthritic mice that were not inoculated with *P*. *gingivalis*. RvD5_n-3 DPA_ administration also upregulated a number of immune-modulatory cytokines, including *Il6*, *Tgfb*, and *Il17a* (Figure 6C), as well as expression of *Tjp1* and *Lyz1* in the intestinal epithelium (Figure 6D). Immunofluorescence analysis of ileal sections demonstrated an increase in E-cadherin expression in arthritic mice inoculated with *P*. *gingivalis* and treated with RvD5_n-3 DPA_ when compared with arthritic mice that were inoculated with the pathobiont and given vehicle only (Figure 6E). Given the role that LMs play in the maintenance of gut homeostasis (27), we next assessed whether RvD5_n-3 DPA_ also regulates intestinal LM profiles. LM profiling demonstrated a shift in the cluster representing profiles obtained with intestinal tissues from arthritic mice inoculated with *P*. *gingivalis* and administered RvD5_n-3 DPA_ away from the cluster of mice given the pathobiont alone. This shift was linked with an increase in the concentrations of several proresolving and tissue-protective mediators, including LXB_4_ and 15-epi-LXB_4_, as well as the n-3 DPA–derived protectin pathway marker 10S,17S-diHDPA (Figure 6F and Supplemental Table 4).

Having observed a rescue of gut barrier function by RvD5_n-3 DPA_, we next investigated whether this was associated with a reduction in joint inflammation. Hence, arthritis was initiated, and mice were inoculated and treated as detailed above. Administration of RvD5_n-3 DPA_ led to a reduction in joint inflammation when compared with vehicle-injected *P*. *gingivalis*–inoculated mice, reaching statistical significance on days 6 (7.0 ± 0.4 versus 12.3 ± 1.9) and 7 (6.5 ± 0.9 versus 10.8 ± 1.5; Figure 6G). Decreased arthritic inflammation was also apparent macroscopically, in terms of reduced joint edema (Figure 6H). Together these results support a role for RvD5_n-3 DPA_ in regulating gut barrier function during inflammatory arthritis, reducing the ability of *P*. *gingivalis* to break down barrier function, thereby preventing exacerbated joint inflammation.

## Discussion

In the present study we demonstrate that inflammatory arthritis alters resolution mechanisms in the gut, weakening barrier function and facilitating barrier breakdown by the pathobiont *P*. *gingivalis*. Using LC-MS/MS–based LM profiling, we found that during inflammatory arthritis, there is a downregulation of several proresolving mediators, including the gut-protective RvD5_n-3 DPA_. This was associated with a shift in macrophage phenotype, downregulation of *Il-10* in the gut tissue, and that of IL-10R and IL-10 in lamina propria macrophages. Furthermore, we found that inflammatory arthritis also leads to a reduction in mucus-producing goblet cell numbers, as well as the expression of tight junction protein *Tjp1* and antimicrobial *Lyz1* in the intestine and an increase in systemic endotoxin concentrations. This loss in intestinal barrier function in arthritic mice was linked with an upregulation of the SPM-inactivating enzyme 15-PGDH and increased further metabolism of RvD5_n-3 DPA_ to its inactive 17-oxo metabolite. This weakening of gut barrier was found to facilitate *P*. *gingivalis*–mediated downregulation of multiple epithelial junction proteins including E-cadherin, which was linked with an increase in bacterial translocation across the gut barrier. *P*. *gingivalis* inoculation also further reduced the production of RvD5_n-3 DPA_ and *Il-10* in the lamina propria and exacerbated arthritic inflammation. Of note, administration of RvD5_n-3 DPA_ to *P*. *gingivalis*–inoculated mice upregulated the expression of *Il-10* in the intestinal epithelium and restored gut barrier function, rectifying host immune responses and reducing joint inflammation.

Loss of gut homeostasis and barrier function is increasingly recognized for its roles in chronic inflammatory disease (43). Given that citrullinated proteins are important autoantibody targets in RA (44), and that the *P*. *gingivalis* peptidyl arginine deiminase (PPAD) enzyme converts arginine peptides to citrulline (39), protein citrullination has been proposed as the mechanism by which *P*. *gingivalis* exacerbates disease in RA. However, a number of conflicting reports put in question the etiological contribution of PPAD to RA (19, 45–47).

Moreover, other, non-citrullinating bacterial species have been reported to exacerbate disease in experimental models of arthritic inflammation (48, 49), suggesting that other mechanisms may be responsible for the observed pathogenesis mediated by *P*. *gingivalis*. Sato and colleagues showed that joint inflammation was worsened by *P*. *gingivalis*, with dysregulated Th17/IL-17A signaling in the collagen-induced arthritis (CIA) model, but found no changes in anti-citrullinated protein responses (19). Aliko et al. and Jeong et al. reported induction of innate immune responses in nonimmune cells by *P*. *gingivalis* (50, 51). The latter found that in vitro incubation of human RA synovial fibroblasts with *P*. *gingivalis* increased expression of TLRs and MMP1 and -3, and IL-8 production (51). In the present study we found that host interaction with *P*. *gingivalis* involves complex signaling networks in mucosal tissues and systemically, and the interactions of heterogenous cell types, including immune cells and microbes. For instance, *P*. *gingivalis* inoculation induced changes in *Tgfb* and *Il17a* expression by mucosal immune cells as well as further downregulating *Tjp1*, *Lyz1*, and E-cadherin in the intestinal epithelium. Importantly, the pathogenic effect of *P*. *gingivalis* was dependent on the dysregulation of the host environment during inflammatory arthritis, such as the decrease in RvD5_n-3 DPA_ levels, gut barrier weakening, and deregulated macrophage phenotype. As the impairment of resolution is a common denominator of chronic inflammatory diseases (6), the self-resolving K/BxN serum transfer model was used to study how induction of inflammatory arthritis may hamper resolution processes. Moreover, human PAD-citrullinated proteins, prominent autoantibody targets in arthritic joints (44), are found across many tissues and in particularly in the gut (52, 53), where they have been proposed to prime autoantigenic responses, which then spread to the joints (52). The K/BxN serum transfer model relies on anti–glucose-6-phosphate isomerase (anti-GPI) antibody–driven inflammatory responses (54). Similar to citrullinated proteins, expression of GPI occurs ubiquitously and has been detected in the gut (55) as well as the joint (56). Intriguingly, one of the proposed mechanisms leading to increased disease severity is a disruption of gut barrier function. Increased gut leakiness has been associated with multiple rheumatic diseases (57), including RA (58). Moreover, a humanized mouse model carrying the human RA susceptibility allele (“shared epitope”) HLA-DRB1*401 showed increased gut permeability prior to the induction of arthritis (59). This study, although it did not provide the underlying mechanism, indicates that increased intestinal permeability can occur during preclinical and early RA due to a genetic predisposition. Results from the present study provide mechanistic insights, as they demonstrate that loss of SPM biosynthesis, including RvD5_n-3 DPA_, is linked with a downregulation in mucus production (Figure 3), thereby exposing the epithelium to intestinal bacteria. In this environment, *P*. *gingivalis* is able to interact with the epithelial lining, downregulating the expression of tight junction molecules including E-cadherin (Figure 1), facilitating barrier breach of microbiota from the gut lumen. This is in line with recent findings in the oral cavity demonstrating that *P*. *gingivalis* expresses extracellular proteases that can degrade junctional adhesion molecules including E-cadherin (60). Future studies will be needed to address whether loss of gut barrier function during inflammatory arthritis may also be induced by autoantibodies to additional antigens, including joint-specific antigens such as type II collagen, in order to determine whether this is a common mechanism of disease propagation in inflammatory arthritis.

Several studies have suggested a link between arthritis and intestinal inflammation in humans (15–17). Macrophages hold a key role in regulating mucosal integrity (61), with recent studies in either IL-10–deficient mice or mice lacking IL-10R specifically on macrophages demonstrating that the IL-10 axis is fundamental in regulating the ability of cells to maintain intestinal epithelial barrier function and that deletion of these proteins leads to spontaneous intestinal inflammation (34, 35). In the present study, we found that the expression of IL-10 was downregulated in both the gut tissue (Figure 1) and lamina propria macrophages (Figure 3) from arthritic mice. This was coupled with a downregulation of IL-10R in lamina propria macrophages (Figure 3). In arthritic mice we also found dysregulation in intestinal epithelium LM concentrations that included a decrease in several proresolving mediators, among which was RvD5_n-3 DPA_ (Figure 2). The regulation of tissue SPM concentrations can occur via multiple mechanisms. In the present study, we found that downregulation of RvD5_n-3 DPA_ was linked with upregulation of 15-PGDH, which converts this mediator to an inactive 17-oxo metabolite (Figure 5). Assessment of the mechanism that leads to the upregulation of this enzyme in macrophages demonstrated that immune complexes, a central part of rheumatic diseases, via the Fc receptors activate ERK1/2, which phosphorylates the transcription factor p-ELK1 (Figure 5), in turn upregulating 15-PGDH expression by binding to Ets-binding sites on the gene promotor (42).

Macrophages play an important role in the biosynthesis of SPMs, and their phenotype is reflected in their mediator profiles (33, 62); in turn, SPMs regulate macrophage phenotype and function (24, 62). Of note, we found that lamina propria macrophages displayed an altered LM profile when compared with cells from naive mice, and this was in part rectified by RvD5_n-3 DPA_, which also upregulated expression of IL-10 and IL-10R on bone marrow–derived macrophages. The role of SPMs in regulating the expression of IL-10 and IL-10R in lamina propria macrophages as well as macrophage phenotype and gut barrier function was supported by findings made in *Alox15*-deficient mice. Here we found that the expression of IL-10 and IL-10R was downregulated on lamina propria macrophages when compared with WT mice (Figure 3). In these mice we also found downregulation in the expression of *Tjp1* and goblet cell function and an increase in systemic endotoxin concentrations. Furthermore, using bone marrow–derived macrophages, we demonstrate that the expression IL-10 and IL-10R was directly regulated by RvD5_n-3 DPA_ via the upregulation of l-kynurenine, a metabolite of tryptophan produced by IDO (Figure 4). This metabolite activates the AHR, which in turn regulates expression of IL-10 and IL-10R by binding to response elements on the promoter regions of their genes (40). Of note, 17-oxo-RvD5_n-3 DPA_ did not increase l-kynurenine concentrations in these macrophages and failed to upregulate expression of either IL-10 and IL-10R (Figure 5). Together, these findings suggest an autocrine role for RvD5_n-3 DPA_ in regulating the expression of the intestinal homeostatic IL-10 axis in lamina propria macrophages. Thus, we hypothesized that disruption in the production of RvD5_n-3 DPA_ would lead to the observed weakening of the gut barrier during inflammatory arthritis, rendering it susceptible to the pathogenic actions of pathobionts such as *P*. *gingivalis*. Indeed, administration of RvD5_n-3 DPA_ to arthritic mice inoculated with *P*. *gingivalis* upregulated the expression of IL-10 in the lamina propria and prevented bacterial barrier breach (Figure 6).

In summary, the present study demonstrates that arthritic inflammation alters intestinal resolution responses, downregulating several protective molecules including RvD5_n-3 DPA_ and dysregulating the expression of the IL-10 axis in both the intestinal epithelium and lamina propria macrophages. This was linked with a disruption in intestinal barrier function that facilitated a switch to pathogenic behavior by the pathobiont *P*. *gingivalis* to further disrupt intestinal resolution processes, promote gut bacterial translocation, and increase joint inflammation. Administration of RvD5_n-3 DPA_ restored gut *Il-10* expression and barrier function, rectified host immune responses, and reduced joint inflammation. Together, these findings have important implications for the management and treatment of RA, in particular in the presence of periodontal disease. Furthermore, they underscore the utility of resolution-based therapeutics for protecting host-microbiota homeostasis in pathobiont-associated diseases.

## Methods

See Supplemental Tables 5–7 for details of primers, antibodies, and reagents used.

### Animals.

Eleven-week-old male specific pathogen–free C57BL/6 mice were procured from Charles River UK. 12/15-Alox-deficient B6.129S2-Alox15^tm1Fun^/J breeder mice were procured from the Jackson Laboratory, then bred in-house. Mice were maintained in individually ventilated cages on a standard chow pellet diet and had access to water ad libitum, with a 12-hour light/12-hour dark cycle.

### Bacterial cultures.

*Porphyromonas gingivalis* W50 (63) and *Bacteroides thetaiotaomicron* E50 (DSM-2079, DSMZ) were grown anaerobically at 37°C on 5% defibrinated horse blood agar (Oxoid/Thermo Fisher) for 72 hours, then in hemin-supplemented (5 μg/mL) brain heart infusion broth (Oxoid/Thermo Fisher) for 48 hours.

### Induction of inflammatory arthritis by K/BxN serum transfer and inoculation with bacteria.

C57BL/6 WT or 12/15-Alox-deficient (12/15-LOX^–/–^) male mice were injected with vehicle (PBS) or arthritogenic K/BxN serum (50 μL, i.p.) on days 0 and 2. Mice were inoculated directly into the stomach by oral gavage with 10^9^ CFU *P*. *gingivalis* W50 (63) or *B*. *thetaiotaomicron* E50 in 100 μL sterile PBS on days –1, 1, and 3. Vehicle control mice were gavaged with 100 μL sterile PBS on the same days. On day 8 after K/BxN serum administration, blood, MLNs, spleens, livers, small and large intestines, paws, and knee joints were collected for further analyses.

For RvD5_n-3 DPA_ treatment,arthritic mice were administered RvD5_n-3 DPA_ (200 ng) in 100 μL saline plus 0.1% ethanol by i.p. injection on days 3 and 5. Clinical scores were assessed (scoring index: sum of inflamed ankles, paws and digits; maximum score, 26/mouse), and paw edema was measured in terms of width of ankles and paws using calipers, to monitor disease development. On termination of the experiments paws, MLNs, spleens, livers, small and large intestines, paws, and knee joints were collected for further analyses.

### LM profiling.

Ice-cold methanol containing 500 pg each of deuterated (d) internal standards (d_8_-5S-hydroxyeicosatetraenoic, d_4_-LTB_4_, d_5_-LXA_4_, d_4_-PGE_2_, and d_5_-RvD2) was added to samples. Spiking with these internal LM standards enables quantification and sample recovery assessment. For joint LM profiling, skin and muscle were removed completely from one hind leg per mouse. For intestinal LM profiling, a piece of the distal ileum or colon (1-cm length) was cut, fecal contents were gently pushed out using curved forceps, and tissues were weighed for normalization. LM extraction and profiling were conducted as described previously (23, 27). In brief, LMs were extracted using an Extrahera (Biotage) autoextractor and solid-phase extraction techniques (23, 27). Next, LC-MS/MS–based LM profiling was used to identify and quantify LMs. Multiple reaction monitoring was conducted using signature Q1 (parent ion) and Q3 (characteristic daughter ion) ion pairs for each molecule, acquired in negative ionization mode. LMs were identified according to published criteria, including matching retention times and with a minimum of 6 diagnostic ions in the tandem mass spectrometry spectra (23, 27). Orthogonal PLS-DA (oPLS-DA) was performed using SIMCA 14.1 software (Umetrics). Details of LMs can be found in Supplemental Table 7.

### qRT-PCR.

Tissues were homogenized in RLT buffer (QIAGEN) containing β-mercaptoethanol (MilliporeSigma) using Lysing Matrix E tubes (MP Biomedicals) and a Precellys 24 tissue homogenizer. Total RNA was isolated from tissues using the RNeasy Mini Kit (QIAGEN) following the manufacturer’s instructions. Total RNA was reverse transcribed using random hexamers or Oligo(dT)20 Primers, dNTPs, RNaseOUT, and SuperScript III reverse transcriptase (all Invitrogen, Thermo Fisher Scientific) and following the manufacturer’s instructions for first-strand cDNA synthesis. Relative quantitative analysis of transcript levels of each gene was performed using PowerUp SYBR Green Master Mix (Thermo Fisher Scientific) and a CFX96 Real-Time System (Bio-Rad) or Applied Biosystems 7900HT Fast Real-Time PCR System (Thermo Fisher Scientific). Target gene expression was normalized against constitutively transcribed housekeeping genes *Gapdh* or *18S* rRNA. Details of primers used are shown in Supplemental Table 5.

### 16S rRNA gene quantitative PCR (16S qPCR).

DNA was isolated from tissues using physical lysis of host tissues and bacterial cells in phenol/chloroform/isoamylalcohol (25:24:1; pH 8.0; MilliporeSigma) (64) using Lysing Matrix E tubes (MP Biomedicals) and a Precellys 24 tissue homogenizer, followed by DNA cleanup using a DNeasy Blood and Tissue kit (QIAGEN). Relative quantitation of bacterial DNA was performed with a PowerUp SYBR Green Master Mix and a CFX96 Real-Time System using primers (16S rRNA) targeting a universal region on the bacterial 16S rRNA gene and normalized against relative levels of eukaryotic DNA in each sample, measured using primers (*gGapdh*) targeting an intron-spanning genomic sequence of *Gapdh*. Primer details can be found in Supplemental Table 5.

### Flow cytometry.

For isolation of lamina propria lymphocytes, small intestines were excised, washed in 2% FBS/HBSS, then cut into small segments. Segments were shaken in 2 mM EDTA/HBSS for 20 minutes at 37°C, then washed with HBSS, followed by further shaking in 2 mM EDTA/HBSS for 20 minutes at 37°C and washing with HBSS. Next, tissues were digested in 1 mg/mL collagenase type VIII (MilliporeSigma) and 10 μg/mL DNase I (MilliporeSigma) in complete RPMI by shaking at 37°C for 35 minutes and passed through 100-μm strainers and single cells centrifuged. Percoll gradient centrifugation was performed to separate leukocytes from other cells, whereby cell pellets were resuspended in 40% Percoll (GE Healthcare) in 2% FBS/RPMI and layered over 75% Percoll in 2% FBS/RPMI. Following centrifugation for 20 minutes, leukocytes concentrated at the interface between the 2 layers were isolated, washed in Dulbecco’s PBS (DPBS) containing 0.02% BSA and 1% Fc-blocking IgG (vol/vol), and incubated in 0.1% live/dead stain for 20 minutes on ice. Cells were fixed with fixation/permeabilization solution (Cytofix/Cytoperm Kit, BD Biosciences) and stained for 30 minutes on ice with fluorescently labeled antibodies (see Supplemental Table 6 for details) first in DPBS containing 0.02% BSA and 1% Fc-blocking IgG (vol/vol) for extracellular staining, then with antibodies in permeabilization solution (Cytofix/Cytoperm Kit) for intracellular staining, following the manufacturer’s instructions.

### Mouse lamina propria–derived macrophage incubations.

Lamina propria lymphocytes were isolated as above and cells incubated in complete PBS-containing calcium and magnesium for 45 minutes on culture plates to allow adhesion. Nonadherent cells were removed by washing. Adherent cells were incubated in complete RPMI with isotype control antibodies or anti-CD16 and anti-CD32 antibodies (see Supplemental Table 6 for details) at 37°C for 20 minutes, then with K/BxN serum (1:1000 dilution) at 37°C for 16 hours. For incubations with RvD5_n-3 DPA_, adherent lamina propria–derived cells were kept incubated with either the SPMs at10 nM or vehicle at 37°C for 20 minutes, followed by incubation with K/BxN serum (1:1000 dilution) at 37°C for 16 hours. Incubations were then quenched with ice-cold methanol, and LMs were identified using LC-MS/MS profiling, as above.

### Mouse bone marrow–derived macrophages.

Bone marrow–derived macrophages were prepared from 10- to 12-week-old C57/BL 6 mice as described previously (65). Macrophages were then seeded into 12-well plates at 2.5 × 10^5^ cells per well and kept overnight in RPMI 1640 containing 10% FBS. Cells were then incubated with PBS, 10 nM RvD5_n-3 DPA_, or 10 nM 17-oxo-RvD5_n-3 DPA_. Supernatants were collected after 2 hours and taken for LC-MS/MS analysis to measure l-kynurenine concentrations. After 16 hours cells were collected, and the expression of IL-10R and IL-10 was assessed using flow cytometry and fluorescently conjugated antibodies as detailed in *Flow cytometry*. In select experiments cells were first incubated with either PBS or INCB 024360-analogfor 30 minutes (37°C), cells were then incubated with 10 nM RvD5_n-3 DPA_, 10 nM 17-oxo-RvD5_n-3 DPA_ or PBS for 16 hours, and the expression of IL-10R and IL-10 was assessed as above.

In separate experiments bone marrow–derived macrophages were incubated with immune complexes or nonspecific IgG for 16 hours, supernatants were collected for LM profiling, and cells were collected for flow cytometry to evaluate the expression of p-ELK1 and 15-PGDH using a rabbit anti–p-ELK1 QDot 800–conjugated antibody and a rabbit anti–15-PGDH Alexa Fluor–conjugated antibody that were prepared using a SiteClick antibody labeling system and APEX antibody labeling system (Thermo Fisher Scientific), respectively. Immunofluorescence staining was performed as described in *Flow cytometry*. To assess the role of ERK1/2 in mediating the upregulation of ELK1 phosphorylation and 15-PGDH expression, we incubated bone marrow–derived macrophages with 20 μM U0126 for 30 minutes at 37°C, cells were then incubated with immune complexes or vehicle for 16 hours at 37°C, and the expression of p-ELK1 and 15-PGDH was evaluated using flow cytometry as above.

### 15-PGDH incubations.

RvD5_n-3 DPA_ (1 μg) was suspended in buffer containing Tris-HCl (100 μL, 50 mM, pH 7.4) and NAD^+^ (1.0 mM) and incubated with 15-PGDH (0.5 U). The incubation was quenched after 30 minutes with ice-cold methanol, and products were taken for C18 solid phase. Products were then identified using LC-MS/MS–based LM profiling as detailed in *LM profiling*. Oxo-containing products were isolated using an Agilent 1270 reversed-phase UV HPLC system equipped with an Agilent Poroshell 120 EC-18 4.6 mm × 100 mm × 2.7 μm column and a diode array detector. Products were eluted with MeOH/H_2_O/acetic acid (70:30:0.01) as phase 1 (*t*_0_, −10 minutes) and a linear gradient with MeOH/acetic acid (99.9:0.1) as phase 2 (10–30 minutes), at a flow rate of 0.5 mL/min.

### Immune complex isolation.

Immune complexes were isolated from K/BxN arthritogenic serum using Melon Gel IgG Purification Kit (Thermo Fisher Scientific) according to the manufacturers’ instructions. In brief, arthritogenic serum was diluted in purification buffer, this was then loaded on to Melon Gel, and fractions with absorbance at 280 nm were collected and pooled. This eluate was then concentrated using 10-kDa centrifugation columns.

### l-Kynurenine quantitation.

Cells were placed in ice-cold MeOH containing d_9_-choline and kept at –20°C for 45 minutes to allow for protein precipitation. Samples were then centrifuged for 10 minutes at 4000 *g*. Supernatants were collected and evaporated under a gentle stream of nitrogen gas using a TurboVap LV (Biotage) at 37°C until dry. Products were then suspended in MeOH and profiled using an LC/MS-MS system. A QTRAP 5500 (AB SCIEX) equipped with a Shimadzu SIL-20AC autoinjector and LC-20AD binary pump was used with an Agilent Eclipse Plus C18 column (100 × 4.6 mm × 1.8 μm). The mobile phase consisted of methanol/water/acetic acid, 80:20:0.01 (vol/vol/vol) for 2.5 minutes, which was ramped to 98:2:0.01 (vol/vol/vol) over 0.2 minutes and maintained for 1.3 minutes. The flow rate was maintained at 0.5 mL/min. To monitor and quantify the levels of l-kynurenine, the QTRAP 5500 was operated in positive mode, and a multiple reaction monitoring (MRM) method was developed with signature ion fragments (*m/z*) for each molecule monitoring the parent ion (Q1) and a daughter ion (Q3). The MRM transition employed for l-kynurenine was 207 > 190 and for d_9_-choline was 113 > 113.

### Limulus amebocyte lysate assay.

Endotoxin levels in plasma were measured using the Pyrochrome Chromogenic Endotoxin test kit (Associates of Cape Cod Inc.) in endpoint mode, according to the manufacturer’s instructions. In brief, 50 μL plasma was incubated on an endotoxin-free 96-well plate with 50μL *Limulus* amebocyte lysate reconstituted in Glucashield buffer for 20–30 minutes at 37°C until distinct coloring was visible in positive control wells. Absorbance was read at a wavelength of 405 nm on a FLUOstar Omega Lite microplate reader (BMG Labtech).

### 16S FISH.

Intestinal tissue was fixed with Carnoy’s fixative (60% methanol, 30% chloroform, 10% glacial acetic acid) and embedded in paraffin, and 5-μm sections were cut. After deparaffinization with xylene and rehydration, a fluorescently labeled probe targeting a universal bacterial 16S rRNA gene sequence ([AminoC6+Alexa488]-GCTGCCTCCCGTAGGAGT-[AmC7~Q+Alexa488]) was used at 10 nM and hybridization performed as described elsewhere (66) to visualize bacteria on intestinal sections. A nonspecific probe ([AminoC6+Alexa488]-ACTCCTACGGGAGGCAGC-[AmC7~Q+Alexa488]) was used as a negative control (66). Hybridized tissues were mounted with DAPI-containing ProLong Gold Antifade mountant (Thermo Fisher Scientific) and visualized with a Zeiss LSM 710 microscope.

### Immunofluorescence staining of intestinal tissue.

After fixation with Carnoy’s fixative (see above) and paraffin embedding of the intestinal tissue, 5-μm sections were cut, deparaffinized with xylene, and rehydrated. This was followed by heat-induced antigen retrieval in 10 mM sodium citrate, pH 6.0. After blocking in 10% normal goat serum, tissues were incubated for 1 hour at room temperature with fluorescently labeled anti–E-cadherin antibody (1:200 in Dako antibody diluent; see Supplemental Table 6 for details). After mounting with DAPI-containing ProLong Gold Antifade mountant, staining was visualized with a Zeiss LSM 710 microscope.

### Periodic acid–Schiff staining.

Intestinal tissue was fixed with Carnoy’s fixative and embedded in paraffin, and sections were cut, then deparaffinized and rehydrated as above. The Merck-Millipore periodic acid–Schiff (PAS) staining kit (VWR) was used according to the manufacturer’s instructions to visualize goblet cells. In short, tissues were stained at room temperature with periodic acid solution for 10 minutes, Schiff’s reagent for 15 minutes, and Mayer’s hematoxylin solution (MilliporeSigma) for 30 seconds, with in-between washing steps with tap water and distilled water. After dehydration in an ascending alcohol series and xylene, sections were mounted with Entellan mountant (Merck) and analyzed using an Olympus BX41 Brightfield light microscope.

### Statistics.

All results are presented as mean ± SEM. Differences between groups were assessed using Mann-Whitney *U* test (2 groups) and 1-way ANOVA (multiple groups) followed by post hoc Dunnett’s test using GraphPad Prism 6 software. Investigators were not blinded to group allocation or outcome assessment. The criterion for statistical significance was *P* ≤ 0.05. Sample sizes for each experiment were determined on the variability observed in prior experiments (67) and preliminary experiments. PLS-DA (68) was performed using SIMCA 14.1 software (Umetrics) following mean centering and unit variance scaling of LM levels. PLS-DA is based on a linear multivariate model that identifies variables that contribute to class separation of observations on the basis of their variables (LM levels). During classification, observations were projected onto their respective class model. The score plot illustrates the systematic clusters among the observations (closer plots presenting higher similarity in the data matrix).

### Study approval.

Approval for animal studies conducted herein was obtained from the United Kingdom Home Office (London, United Kingdom), in strict accordance with Home Office regulations (Guidance on the Operation of Animals [Scientific Procedures] Act) and Laboratory Animal Science Association Guidelines (*Guiding Principles on Good Practice for Animal Welfare and Ethical Review Bodies*).

## Author contributions

MBF and JD designed the experiments and conceived the overall research plan. MBF, RAC, JD, and EMA conducted the experiments and analyzed results. MBF and JD wrote the manuscript. All authors contributed to manuscript preparation. CP, MAC, and JD contributed to supervision of the work.

## Supplementary Material

Supplemental data

## Version 1 07/11/2019

Electronic publication
