# Inflammatory arthritis disrupts gut resolution mechanisms, promoting barrier breakdown by ﻿*Porphyromonas﻿﻿ ﻿gingivalis*

**DOI:** 10.1172/jci.insight.165600

**Published:** 2022-10-24

**Authors:** Magdalena B. Flak, Romain A. Colas, Estefanía Muñoz-Atienza, Michael A. Curtis, Jesmond Dalli, Costantino Pitzalis

Original citation: *JCI Insight*. 2019;4(13):e125191. https://doi.org/10.1172/jci.insight.125191

Citation for this corrigendum: *JCI Insight*. 2022;7(20):e165600. https://doi.org/10.1172/jci.insight.165600

The authors recently became aware that representative illustrations presented in Figure 2A might be mistaken for original data. As this panel was used strictly for demonstrative purposes, it has been removed from the figure for clarity. The updated figure, figure legend, and description in the Results section are below.

## Results

Using liquid chromatography–tandem mass spectrometry–based (LC-MS/MS–based) LM profiling, we identified mediators from all 4 major fatty acid bioactive metabolomes, including lipoxygenase- and cyclooxygenase-derived LMs that were identified in accordance with published criteria (33). The identity of each of the mediators was further corroborated by evaluating MS/MS spectra obtained in a subset of the samples analyzed and matching at least 6 diagnostic ions with those obtained for reference standards for each of these molecules, as shown for RvD5_n-3 DPA_ (Figure 2A) (23). Multivariate analysis of identified mediators gave 2 distinct clusters, demonstrating a marked shift in intestinal LM concentrations in arthritic mice (Figure 2B). This shift was linked with a reduction in proresolving mediator concentrations in arthritic mice. Among the mediator families that were downregulated, we obtained a significant reduction in the recently uncovered n-3 DPA–derived resolvins (RvD_n-3 DPA_), including the gut-protective RvD5_n-3 DPA_ (27) (Figure 2, B–D, and Supplemental Table 1).

## Figures and Tables

**Figure 2:**
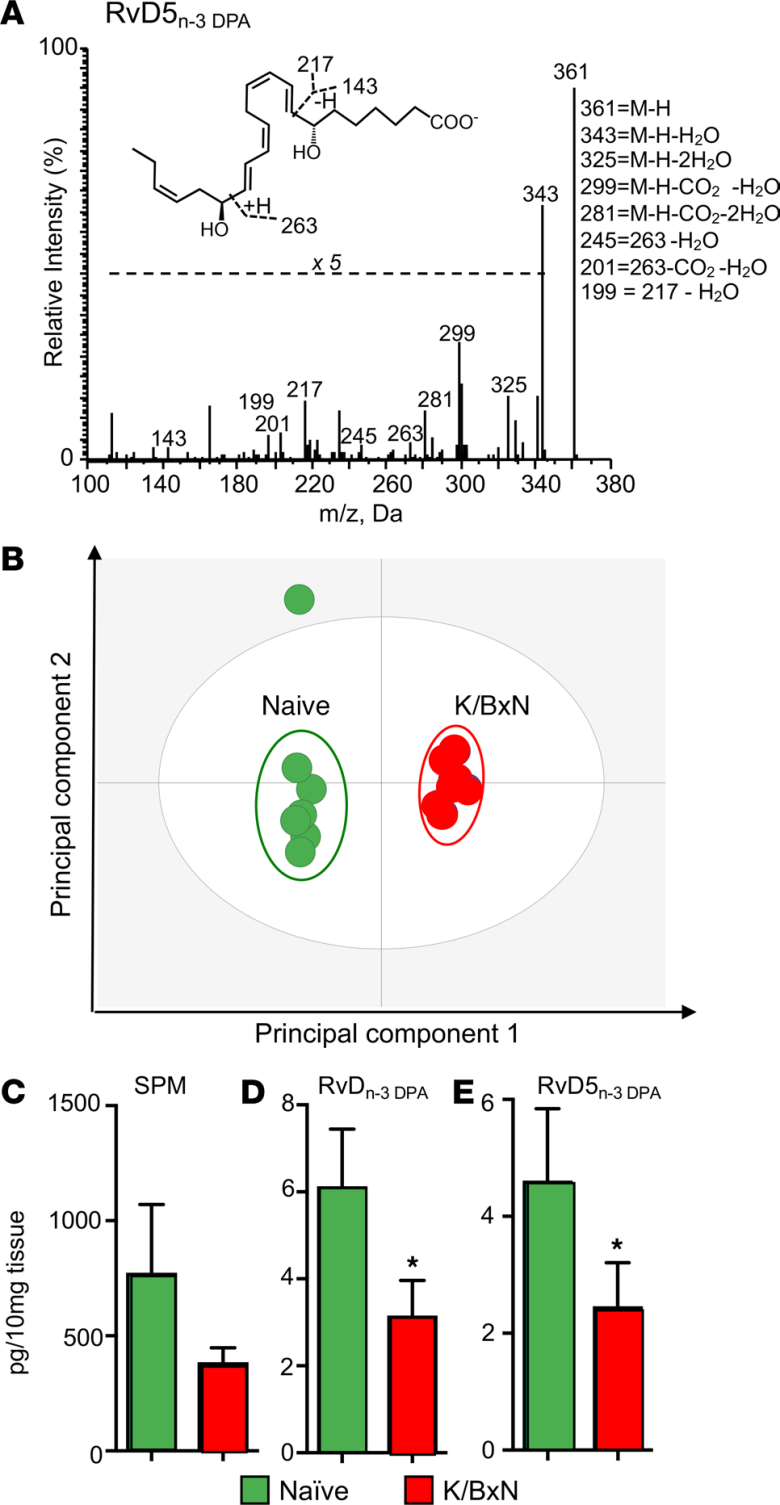
Arthritis dysregulates intestinal lipid mediator profiles. Arthritis was initiated by injection of K/BxN serum (50 μL per mouse, i.p.; days 0 and 2). On day 8, ilea were harvested from arthritic and naive mice and lipid mediators identified and quantified using lipid mediator profiling (see Methods for details). (**A**) MS/MS spectrum employed for the identification of RvD5_n-3 DPA_. Labeled ions are those matching assigned ion fragments for RvD5_n-3 DPA_. Inset, portion of the molecule that corresponds to each of the diagnostic ions; M, molecular mass. (**B**) Orthogonal partial least squares discriminant analysis (oPLS-DA) of intestinal lipid mediator profiles. Cumulative tissue concentrations for SPMs (i.e., arachidonic-, eicosapentaenoic acid–, n-3 docosapentaenoic– [DPA–], and docosahexaenoic acid–derived [DHA-derived] proresolving mediators) (**C**), RvD_n-3 DPA_ (i.e., RvD1_n-3 DPA_, RvD2_n-3 DPA_, and RvD5_n-3 DPA_) (**D**), and RvD5_n-3 DPA_ (**E**). Results for **A** are representative of *n* = 24 mice; for **B** are representative of *n* = 8 mice per group; for **C**–**E** are mean ± SEM for *n* = 8 mice per group from 2 independent experiments; *P ≤ 0.05 versus naive using Mann-Whitney *U* test. Results are expressed as pg/10 mg tissue.

## Version 1 10/24/2022

Electronic publication

