# Supplementary material for: Inflammatory arthritis disrupts gut resolution mechanisms, promoting barrier breakdown by Porphyromonas gingivalis
Source: JCI Insight. 2019 Jul 11;4(13):e125191. doi: 10.1172/jci.insight.125191 (PMC6629160; doi:10.1172/jci.insight.125191)

Title:

Inflammatory arthritis disrupts gut resolution mechanisms promoting barrier breakdown by *Porphyromonas gingivalis*

Authors:

Magdalena B. Flak[1], Romain A. Colas[2], Estefanía Muñoz-Atienza[1], Michael A. Curtis[3], Jesmond Dalli[2,4,#], Costantino Pitzalis[1,4,#]

[1]Centre for Experimental Medicine & Rheumatology, William Harvey Research Institute, QMUL, London EC1M 6BQ, U.K.;

[2]Lipid Mediator Unit, William Harvey Research Institute, QMUL, London EC1M 6BQ, U.K.;

[3]Dental Institute, King's College London, London SE1 9RT, U.K;

[4]Centre for Inflammation and Therapeutic Innovation, Queen Mary University of London, London, UK.

# - share senior authorship

Corresponding authors: Dr Magdalena B. Flak: m.b.flak@qmul.ac.uk

Dr Jesmond Dalli: j.dalli@qmul.ac.uk

Supplemental materials
Supplemental Tables
Supplemental Table 1: Inflammatory arthritis dysregulates ileal SPM concentrations

			Ileal lipid mediator concentration (pg/mL)					
**DHA bioactive metabolome**	**Q1**	**Q3**	**Naïve**			**K/BxN + Vehicle**		
RvD1	375	141	3.8	±	3.3	0.4	±	0.1
RvD2	375	141	73.6	±	77.2	1.6	±	0.7
RvD3	375	147	0.2	±	0.1		-	*
RvD4	375	101	44.6	±	15.8	1.5	±	0.6 *
RvD5	359	199	9.7	±	4.4	5.9	±	1.7
RvD6	359	101	5.8	±	3.7	1.9	±	0.8
17R-RvD1	375	141	6.9	±	5.5	0.2	±	0.1
17R-RvD3	375	147	0.6	±	0.5	0.2	±	0.1
PD1	359	153	35.4	±	12.6	6.0	±	1.8 *
10S,17S-diHDHA	359	153	39.3	±	12.8	49.8	±	10.1
17R-PD1	359	153	19.1	±	10.2	4.7	±	1.1
22-OH-PD1	375	153	0.6	±	0.4	0.2	±	0.1
MaR1	359	221	0.7	±	0.3	0.3	±	0.2
MaR2	359	221	6.9	±	3.7	5.6	±	1.3
7S,14S-diHDHA	359	221	0.2	±	0.1	0.2	±	0.1
22-OH-MaR1	375	221		-			-	
4S,14S-diHDHA	359	101	1.3	±	0.5	2.3	±	0.8
**n-3 DPA bioactive metabolome**								
$RvD1_{n-3\ DPA}$	377	143	0.9	±	0.3	0.4	±	0.2
$RvD2_{n-3\ DPA}$	377	261	0.6	±	0.3	0.3	±	0.2
$RvD5_{n-3\ DPA}$	361	263	4.6	±	1.3	2.5	±	0.5 *
RvT1	377	193	1.3	±	0.7	0.8	±	0.5
RvT2	377	143	2.5	±	2.2	0.1	±	0.1
RvT3	377	255	3.5	±	2.0	1.7	±	0.3
RvT4	359	193	5.2	±	2.7	1.1	±	0.3
$PD1_{n-3\ DPA}$	361	183	12.7	±	3.6	37.0	±	8.1
10S, 17S-diHDPA	377	255	115.3	±	121.7	205.5	±	64.5
$MaR1_{n-3\ DPA}$	361	249	2.2	±	1.0	1.8	±	0.5
**EPA bioactive metabolome**								
RvE1	349	195	2.5	±	2.4	0.2	±	0.1
RvE2	333	199	3.8	±	2.0	0.7	±	0.4
RvE3	333	201	12.6	±	4.3	1.4	±	0.5 **
**AA bioactive metabolome**								
LXA_4	351	217	1.0	±	0.4	0.2	±	0.1 *
LXB_4	351	221	45.7	±	29.4	6.9	±	1.5
5S,15S-diHETE	335	235	191.5	±	36.3	40.4	±	22.0 **
15epi-LXA_4	351	217	79.9	±	24.8	1.4	±	0.4 **
15epi-LXB_4	351	221	7.5	±	6.2	0.8	±	0.2
LTB_4	335	195	7.0	±	2.0	3.2	±	0.6 *
5S,12S-diHETE	335	195	2.6	±	1.1	1.5	±	0.3
Δ6-trans, 12-epi-LTB_4	351	195	9.9	±	4.0	1.7	±	0.3 *
Δ6-trans-LTB_4	351	195	17.3	±	7.2	2.3	±	0.7 *
20-OH-LTB_4	351	195	0.3	±	0.1	0.2	±	0.2
PGD_2	351	189	1946.7	±	407.5	1080.5	±	575.3
PGE_2	351	189	4085.9	±	517.5	1815.3	±	898.4 *
$PGF_{2\alpha}$	353	193	800.9	±	239.5	419.3	±	173.4
TxB_2	369	169	778.5	±	199.4	220.3	±	70.9 **

Inflammatory arthritis in mice was initiated by injection of K/BxN serum (50μl per mouse, *i.p.*; Days 0, 2). On day 8 ilea were harvested and lipid mediators identified and quantified using lipid mediator (LM) profiling (see Methods for details). Results are mean ± SEM; n = 8 mice per group. -, below limit, limit ≈ 0.1 pg. *p<0.05, **p<0.01 *versus* naïve mice.

Supplemental Table 2: Arthritogenic factors dysregulate SPM production by lamina propria macrophages.

			Lamina propria macrophage lipid mediator concentration (pg/mL)								
**DHA bioactive metabolome**	**Q1**	**Q3**	**Naïve**			**K/BxN + Isotype Ab**			**Anti-CD16/CD32 + K/BxN**		
RvD1	375	141	4.41	±	1.19	7.56	±	3.61	8.19	±	4.73
RvD2	375	141	0.15	±	0.11	0.12	±	0.08	0.07	±	0.04
RvD3	375	147	0.54	±	0.08	0.39	±	0.13	0.48	±	0.02
RvD4	375	101	4.81	±	1.34	10.01	±	2.74 *	7.64	±	1.73 #
RvD5	359	199	1.45	±	0.45	1.10	±	0.19	1.48	±	0.22
RvD6	359	101	0.25	±	0.11	0.18	±	0.09	0.24	±	0.08
17R-RvD1	375	141	1.38	±	1.14	0.54	±	0.36	0.32	±	0.14
17R-RvD3	375	147	1.15	±	0.77	0.85	±	0.41	0.77	±	0.24
PD1	359	153	1.31	±	0.40	2.15	±	0.84	2.19	±	1.03
10S,17S-diHDHA	359	153	15.98	±	2.97	15.69	±	4.95	14.88	±	5.67
17R-PD1	359	153	1.21	±	0.35	0.87	±	0.32	1.40	±	0.69
22-OH-PD1	375	153	0.47	±	0.49	0.15	±	0.03	0.09	±	0.06
MaR1	359	221	2.08	±	0.88	0.98	±	0.29	1.76	±	0.86
MaR2	359	221	0.82	±	0.64	0.45	±	0.12	0.36	±	0.07
7S,14S-diHDHA	359	221	9.72	±	8.63	4.08	±	1.89	3.64	±	0.66
22-OH-MaR1	375	221	2.36	±	2.26	0.36	±	0.08	0.28	±	0.06
4S,14S-diHDHA	359	101	1.61	±	0.72	1.91	±	0.29	2.23	±	0.61
**n-3 DPA bioactive metabolome**											
$RvD1_{n\text{-}3\ DPA}$	377	143	0.50	±	0.19	0.84	±	0.21	0.39	±	0.14 #
$RvD2_{n\text{-}3\ DPA}$	377	261	0.63	±	0.28	0.84	±	0.35 *	0.32	±	0.15
$RvD5_{n\text{-}3\ DPA}$	361	263	1.27	±	0.36	0.49	±	0.10 *	0.88	±	0.21
RvT1	377	193	0.53	±	0.39	0.11	±	0.07	0.12	±	0.06
RvT2	377	143	1.48	±	0.48	1.21	±	0.28	1.43	±	0.58
RvT3	377	255	0.05	±	0.03	0.06	±	0.03	0.04	±	0.02
RvT4	359	193	0.87	±	0.47	0.38	±	0.09	0.53	±	0.13
$PD1_{n\text{-}3\ DPA}$	361	183	0.10	±	0.11	0.12	±	0.04	0.14	±	0.03
10S, 17S-diHDPA	361	183	0.81	±	0.24	1.22	±	0.42	0.77	±	0.26
$PD2_{n\text{-}3\ DPA}$	361	233	0.39	±	0.10	0.75	±	0.36	0.63	±	0.32 #
$MaR1_{n\text{-}3\ DPA}$	361	223	0.00	±	0.00	0.00	±	0.00	0.00	±	0.00
7S, 14S-diHDPA	361	223	0.67	±	0.34	0.35	±	0.16	0.21	±	0.07
$MaR2_{n\text{-}3\ DPA}$	361	193	16.56	±	6.92	14.25	±	5.01	11.66	±	3.36
**EPA bioactive metabolome**											
RvE1	349	195	0.68	±	0.38	2.06	±	0.63 *	1.76	±	0.50
RvE2	333	199	0.17	±	0.11	0.43	±	0.09 *	0.57	±	0.19
RvE3	333	201	0.12	±	0.09	0.36	±	0.11 *	0.88	±	0.24
**AA bioactive metabolome**											
LXA_4	351	115	0.41	±	0.28	0.21	±	0.04	0.08	±	0.03
LXB_4	351	221	12.38	±	1.65	3.34	±	1.57 *	3.30	±	1.49
5S,15S-diHETE	335	235	54.76	±	5.68	49.74	±	4.99	48.25	±	5.95
15epi-LXA_4	351	115	8.36	±	1.47	12.49	±	1.82 *	12.87	±	1.41
15epi-LXB_4	351	115	0.25	±	0.12	0.12	±	0.04	0.29	±	0.03 #
13,14-dihydro, 15-oxo-LXA_4	351	115	1.04	±	0.25	0.58	±	0.16 *	0.51	±	0.06
LTB_4	335	195	5.05	±	1.17	6.18	±	1.04	5.97	±	1.29
5S,12S-diHETE	335	195	0.00	±	0.00	0.00	±	0.00	0.00	±	0.00
20-OH-LTB_4	351	195	0.03	±	0.04	0.01	±	0.01	0.02	±	0.01
PGD_2	351	189	291.74	±	70.21	998.89	±	30.74 *	513.61	±	58.23 #
PGE_2	351	189	889.28	±	66.21	1295.39	±	14.28 *	944.28	±	95.92 #
$PGF_{2\alpha}$	353	193	194.06	±	24.25	278.65	±	18.95 *	220.81	±	41.14 #
TxB_2	369	169	846.74	±	204.04	949.12	±	79.39	475.28	±	58.58 #

Macrophages were isolated from the lamina propria of naïve mice and incubated with isotype control antibody (Isotype Ab), anti-Fc antibody (anti-CD16/CD32) or vehicle (15 min, 37°C), then with K/BxN serum or PBS (16h, 37°C). Incubations were quenched using 2 volumes of ice-cold methanol and lipid mediators were identified and quantified using LM profiling (see Methods for details). Results are mean ± SEM; n = 4 donor mice per group. -, below limit, limit ≈ 0.1 pg. *$p<0.05$ *versus* naïve macrophages; #$p<0.05$ *versus* K/BxN-treated macrophages.

Supplemental Table 3: RvD5$_{n-3\ DPA}$ regulates eicosanoid production by lamina propria macrophages from arthritic mice.

			Lamina propria macrophage lipid mediators concentration (pg/mL)								
**DHA bioactive metabolome**	**Q1**	**Q3**	**Naïve**			**K/BxN**			**K/BxN+ RvD5$_{n-3\ DPA}$**		
RvD1	375	141	4.41	±	1.19	18.86	±	7.25 *	20.57	±	6.45
RvD2	375	141	0.15	±	0.11	0.10	±	0.12	0.19	±	0.14
RvD3	375	147	0.54	±	0.08	0.26	±	0.19	0.25	±	0.12
RvD4	375	101	4.81	±	1.34	6.25	±	1.91	6.17	±	0.51
RvD5	359	199	1.45	±	0.45	3.48	±	1.34 *	3.73	±	1.24
RvD6	359	101	0.25	±	0.11	0.20	±	0.14	0.31	±	0.17
17R-RvD1	375	141	1.38	±	1.14	0.44	±	0.51	0.75	±	0.85
17R-RvD3	375	147	1.15	±	0.77	0.76	±	0.44	0.64	±	0.51
PD1	359	153	1.31	±	0.40	2.12	±	1.40	25.26	±	27.47
10S,17S-diHDHA	359	153	15.98	±	2.97	27.74	±	11.62	31.04	±	9.46
17R-PD1	359	153	1.21	±	0.35	2.22	±	1.17	2.56	±	1.09
22-OH-PD1	375	153	0.47	±	0.49	0.37	±	0.17	0.56	±	0.45
MaR1	359	221	2.08	±	0.88	2.54	±	1.71	1.84	±	0.92
MaR2	359	221	0.82	±	0.64	2.73	±	1.75	4.64	±	1.98
7S,14S-diHDHA	359	221	9.72	±	8.63	3.39	±	1.13	2.91	±	0.91
22-OH-MaR1	375	221	2.36	±	2.26	0.57	±	0.79	0.91	±	0.54
4S,14S-diHDHA	359	101	1.61	±	0.72	3.36	±	1.12	3.91	±	1.21
**n-3 DPA bioactive metabolome**											
RvD1$_{n-3\ DPA}$	377	143	0.50	±	0.19	2.55	±	1.14 *	2.17	±	0.66
RvD2$_{n-3\ DPA}$	377	261	0.63	±	0.28	0.39	±	0.18	0.83	±	0.61
RvD5$_{n-3\ DPA}$	361	143	4.6	±	1.3	0.87	±	0.33	212.70	±	142.45 * #
RvT1	377	193	0.53	±	0.39	1.12	±	0.91	0.39	±	0.22
RvT2	377	143	1.48	±	0.48	2.96	±	1.18	7.34	±	2.46 #
RvT3	377	255	0.05	±	0.03	0.00	±	0.00	0.02	±	0.04
RvT4	359	193	0.87	±	0.47	0.59	±	0.13	0.59	±	0.23
PD1$_{n-3\ DPA}$	361	183	0.10	±	0.11	0.12	±	0.07	0.14	±	0.11
10S, 17S-diHDPA	361	183	0.81	±	0.24	2.39	±	0.56 *	11.31	±	4.75 #
PD2$_{n-3\ DPA}$	361	233	0.39	±	0.10	3.09	±	0.69 *	4.42	±	0.87 #
MaR1$_{n-3\ DPA}$	361	223	0.00	±	0.00	0.00	±	0.00	0.00	±	0.00
7S, 14S-diHDPA	361	223	0.67	±	0.34	0.71	±	0.17	1.16	±	0.31 #
MaR2$_{n-3\ DPA}$	361	193	16.56	±	6.92	12.57	±	2.01	11.96	±	1.80 #
**EPA bioactive metabolome**											
RvE1	349	195	0.68	±	0.38	17.40	±	8.77 *	15.78	±	6.68
RvE2	333	199	0.17	±	0.11	0.29	±	0.39	0.41	±	0.52
RvE3	333	201	0.12	±	0.09	0.41	±	0.29	0.47	±	0.47
**AA bioactive metabolome**											
LXA$_4$	351	115	0.41	±	0.28	0.25	±	0.14	0.27	±	0.19
LXB$_4$	351	221	12.38	±	1.65	9.10	±	5.60	10.03	±	2.59
5S,15S-diHETE	335	235	54.76	±	5.68	53.26	±	13.39	59.90	±	3.36
15epi-LXA$_4$	351	115	8.36	±	1.47	13.27	±	3.12	11.36	±	2.37 #
15epi-LXB$_4$	351	115	0.25	±	0.12	0.22	±	0.12	0.54	±	0.15 #
13,14-dihydro, 15-oxo-LXA$_4$	351	115	1.04	±	0.25	2.46	±	0.30	2.13	±	0.41
LTB$_4$	335	195	5.05	±	1.17	9.02	±	1.21 *	7.74	±	0.73
5S,12S-diHETE	335	195	0.00	±	0.00	0.00	±	0.00	0.00	±	0.00
20-OH-LTB$_4$	351	195	0.03	±	0.04	0.11	±	0.23	0.21	±	0.41
PGD$_2$	351	189	291.74	±	70.21	3444.05	±	737.88 **	2577.41	±	386.27 #
PGE$_2$	351	189	889.28	±	66.21	4342.17	±	1135.24 **	2634.38	±	417.90 #
PGF$_{2\alpha}$	353	193	194.06	±	24.25	1087.66	±	289.94 **	1123.14	±	328.79
TxB$_2$	369	169	846.74	±	204.04	4606.45	±	958.14 **	2889.10	±	564.59 #

Macrophages were isolated from the lamina propria of naïve (Naïve) and arthritogenic mice and incubated with 10nM $RVD5_{n-3\ DPA}$ (K/BxN + $RVD5_{n-3\ DPA}$) or vehicle (K/BxN; 16h, 37°C). Incubations were quenched using 2 volumes of ice-cold methanol and lipid mediators were identified and quantified using LM profiling (see Methods for details). Results are mean ± SEM; n = 4 donor mice per group. -, below limit, limit ≈ 0.1 pg. *p<0.05, ** p<0.001 *versus* naïve macrophages; #p<0.05 *versus* K/BxN-treated macrophages.

Supplemental Table 4: RvD5$_{n\text{-}3\ DPA}$ upregulates ileal SPM concentrations in *P. gingivalis*-inoculated arthritic mice.

			Ileal lipid mediator concentration (pg/mL)		
**DHA bioactive metabolome**	**Q1**	**Q3**	**K/BxN + Vehicle**	**K/BxN + *P. gingivalis***	**K/BxN +*P.gingivalis* + RvD5$_{n\text{-}3\ DPA}$**
RvD1	375	141	0.4 ± 0.1	0.1 ± 0.0 *	0.2 ± 0.1
RvD2	375	141	1.6 ± 0.7	0.4 ± 0.2 *	0.6 ± 0.1 #
RvD3	375	147	0.0 ± 0.0	0.2 ± 0.1 *	0.3 ± 0.2 #
RvD4	375	101	1.5 ± 0.6	2.2 ± 0.6	2.5 ± 1.1
RvD5	359	199	5.9 ± 1.7	8.9 ± 2.6	14.5 ± 5.4
RvD6	359	101	1.9 ± 0.8	1.9 ± 0.3	2.3 ± 0.5
17R-RvD1	375	141	0.2 ± 0.1	- *	0.1 ± 0.1
17R-RvD3	375	147	0.2 ± 0.1	0.2 ± 0.1	0.2 ± 0.1
PD1	359	153	6.0 ± 1.8	7.5 ± 1.6	6.8 ± 1.8
10S,17S-diHDHA	359	153	49.8 ± 10.1	33.3 ± 9.1	38.7 ± 7.3
17R-PD1	359	153	4.7 ± 1.1	9.2 ± 6.5	4.5 ± 1.1
22-OH-PD1	375	153	0.2 ± 0.1	0.0 ± 0.0	0.0 ± 0.0
MaR1	359	221	0.3 ± 0.2	0.4 ± 0.2	0.2 ± 0.1
MaR2	359	221	5.6 ± 1.3	2.4 ± 0.7 *	2.1 ± 0.9 #
7S,14S-diHDHA	359	221	0.2 ± 0.1	0.1 ± 0.1	0.2 ± 0.2
22-OH-MaR1	375	221	0.4 ± 0.4	0.3 ± 0.3	0.8 ± 0.3
4S,14S-diHDHA	359	101	2.3 ± 0.8	0.6 ± 0.3 *	1.2 ± 0.3
**n-3 DPA bioactive metabolome**					
RvD1$_{n\text{-}3\ DPA}$	377	143	0.4 ± 0.2	0.3 ± 0.1	0.3 ± 0.2
RvD2$_{n\text{-}3\ DPA}$	377	261	0.3 ± 0.2	0.4 ± 0.2	0.3 ± 0.2
RvD5$_{n\text{-}3\ DPA}$	361	263	2.5 ± 0.8	0.7 ± 0.2 *	2.5 ± 1.6
RvT1	377	193	0.8 ± 0.5	0.9 ± 0.6	1.2 ± 0.5
RvT2	377	143	0.1 ± 0.1	0.1 ± 0.1	0.1 ± 0.1
RvT3	377	255	1.7 ± 0.3	1.5 ± 0.2	1.6 ± 0.5
RvT4	359	193	1.1 ± 0.3	1.1 ± 0.3	2.1 ± 1.0
PD1$_{n\text{-}3\ DPA}$	361	183	37.0 ± 8.1	25.0 ± 10.5	38.3 ± 8.0
10S, 17S-diHDPA	377	255	205.5 ± 64.5	52.8 ± 16.7	133.6 ± 29.2 #
MaR1$_{n\text{-}3\ DPA}$	361	249	1.8 ± 0.5	1.1 ± 0.5	4.1 ± 2.9
**EPA bioactive metabolome**					
RvE1	349	195	0.2 ± 0.1	0.2 ± 0.1	0.2 ± 0.1
RvE2	333	199	0.7 ± 0.4	1.9 ± 0.8	1.8 ± 0.6
RvE3	333	201	1.4 ± 0.5	1.0 ± 0.3	1.8 ± 0.6
**AA bioactive metabolome**					
LXA_4	351	217	0.2 ± 0.1	0.3 ± 0.0	0.3 ± 0.1
LXB_4	351	221	6.9 ± 1.5	12.1 ± 2.1	23.4 ± 6.8 * #
5S,15S-diHETE	335	235	40.4 ± 22.0	19.8 ± 4.0	44.6 ± 19.1
15epi-LXA_4	351	217	1.4 ± 0.4	1.5 ± 0.2	2.9 ± 0.2 #
15epi-LXB_4	351	221	0.8 ± 0.2	1.3 ± 0.3	0.6 ± 0.2
LTB_4	335	195	3.2 ± 0.6	3.0 ± 1.3	1.9 ± 0.1 *
5S,12S-diHETE	335	195	1.5 ± 0.3	1.3 ± 0.3	0.7 ± 0.1 *
Δ6-trans, 12-epi-LTB_4	351	195	1.7 ± 0.3	1.4 ± 0.6	1.0 ± 0.1 *
Δ6-trans-LTB_4	351	195	2.3 ± 0.7	1.6 ± 0.5	1.6 ± 0.1
20-OH-LTB_4	351	195	0.2 ± 0.2	0.6 ± 0.5	0.1 ± 0.1
PGD_2	351	189	1080.5 ± 575.3	1353.5 ± 616.6	1107.3± 279.4
PGE_2	351	189	1815.3 ± 898.4	2244.3 ± 839.1	2890.1± 570.4 *
$PGF_{2\alpha}$	353	193	419.3 ± 173.4	321.2 ± 113.4	379.2 ± 93.8
TxB_2	369	169	220.3 ± 70.9	201.5 ± 79.6	226.6 ± 73.5

Mice were inoculated with *P. gingivalis* (10^9 CFU per mouse; Days -1, 1, 3) and arthritis initiated by injection of K/BxN serum (50µl per mouse, i.p.; Days 0, 2). On days 3 and 5 mice were given 200ng/mouse $RvD5_{n\text{-}3\ DPA}$ or Vehicle (PBS+0.01% EtOH) and on day 8 ilea were harvested and lipid mediators identified and quantified using LM profiling (see Methods for details). Results are mean ± SEM; n = 8 mice per group. -, below limit, limit ≈ 0.1 pg. *p<0.05, *versus* K/BxN mice; [#]p<0.05 *versus* K/BxN + *P. gingivalis* mice.

Supplemental Table 5: Oligonucleotides

OLIGONUCLEOTIDES	SOURCE	IDENTIFIER
Gapdh	Invitrogen, UK; Custom Oligos	forward: 5'-CATGTTCCAGTATGACTCCA-3', reverse: 5'-TGAAGACACCAGTAGACTCC-3'
18S rRNA	Invitrogen, UK; Custom Oligos	forward: 5'-CATTCGAACGTCTGCCCTATC -3' reverse: 5'-CCTGTGCCTTCCTTGGA-3'
Tjp-1	Invitrogen, UK; Custom Oligos	forward: 5'-ACCACCAACCCGAGAAGAC-3' reverse: 5'-CAGGAGTCATGGACGCACA-3'
Lyz1	Invitrogen, UK; Custom Oligos	forward: 5'-GCCAAGGTCTACAATCGTTGTGAGTTG-3' reverse: 5'-CAGTCAGCCAGCTTGACACCACG-3'
Il-17a	Invitrogen, UK; Custom Oligos	forward: 5'-GCTCCAGAAGGCCCTCAGA-3' reverse: 5'-CTTTCCCTCCGCATTGACA-3'
Il-10	Invitrogen, UK; Custom Oligos	forward: 5'-GAGAGCTGCAGGGCCCTTTGC-3' reverse: 5'-CTCCCTGGTTTCTCTTCCCAAGACC-3'
Tgfb1	Invitrogen, UK; Custom Oligos	forward: 5'-TGTACGGCAGTGGCTGAACCA-3' reverse: 5'-TGTCACAAGAGCAGTGAGCGCT-3'
Il-6	Invitrogen, UK; Custom Oligos	forward: 5'-TAGTCCTTCCTACCCCAATTTCC-3' reverse: 5'-TTGGTCCTTAGCCACTCCTTC-3'
Kc	Invitrogen, UK; Custom Oligos	forward: 5'-CCGAAGTCATAGCCACACTCAA-3' reverse: 5'-GCAGTCTGTCTTCTTTCTCCGTTAC-3'

gGapdh	Invitrogen, UK; Custom Oligos	forward: 5’-ACATCATCCCTGCCTCTAC-3’ reverse: 5’-TCAAAGGTGGAGGAGTGG-3’
Il-23a; Mm_Il23a_2_SG QuantiTect Primer Assay	Qiagen, UK	Cat # QT01663613
Rorc; Mm_Rorc_1_SG QuantiTect Primer Assay	Qiagen, UK	Cat # QT00197722
Muc2; Mm_Muc2_2_SG QuantiTect Primer Assay	Qiagen, UK	Cat # QT01060773
Il-10ra; Mm_Il10ra_1_SG QuantiTect Primer Assay	Qiagen, UK	Cat # QT00112742
genomic Gapdh	PMID: 16619041 Invitrogen, UK; Custom Oligos	forward: 5’-CATGTTCCAGTATGACTCCA-3’ reverse: 5’-TGAAGACACCAGTAGACTCC-3’
16S rRNA	PMID: 21998396; Invitrogen, UK; Custom Oligos	forward: 5’-ACTCCTACGGGAGGCAGCAGT-3’ reverse: 5’-ATTACCGCGGCTGCTGGC-3’
Alexa488-labelled FISH probe,16S	PMID: 21998396; Custom Oligo, Eurofins Genomics, UK	[AminoC6 +Alexa488]-GCTGCCTCCCGTAGGAGT-[AmC7~Q +Alexa488]
Alexa488-labelled FISH probe, NS Ctrl	PMID: 21998396; Custom Oligo, Eurofins Genomics, UK	([AminoC6 +Alexa488]-ACTCCTACGGGAGGCAGC-[AmC7~Q +Alexa488])

Supplemental Table 6: Antibodies

ANTIBODIES	SOURCE	IDENTIFIER
APC/Cy7 anti-mouse CD45	Biolegend	Clone 30-F11; Cat # 103116
Alexa Fluor 488 anti-mouse/human CD11b	Biolegend	Clone M1/70; Cat # 101217
Alexa Fluor 700 anti-mouse Ly-6G	Biolegend	Clone 1A8; Cat # 127622
PE anti-mouse CD64 (FcγRI)	Biolegend	Clone X54-5/7.1 Cat # 139304
Brilliant Violet 785™ anti-mouse Ly-6C	Biolegend	Clone HK1.4 Cat # 128041
BV510 anti-mouse CD43	BD Biosciences	Clone S7 Cat # 563206
APC anti-mouse SiglecF	Miltenyi Biotec	Clone ES22-10D8 Cat # 130-102-241
Fc-blocking IgG (anti-mouse CD16/32)	Biolegend	Clone 93 Cat # 101310
PE/Dazzle 594 Rat Anti-Mouse IL-10	Biolegend	Clone JES5-16E3 Cat # 505033
PE anti-mouse CD210 (IL-10 R)	Biolegend	Clone 1B1.3a Cat # 112705
Alexa Fluor® 594 anti-mouse/human CD324 (E-Cadherin)	Biolegend	Clone DECMA-1 Cat # 147306
PE-Dazzle 594 anti-Human CX3CR1	Biolegend	Clone 2A9-1 Cat # 341623
Alexa Fluor 488 Rabbit mAb Anti-Human/Mouse/Rat COX-2 (D5H5) XP	Cell Signaling	Cat # 13596S
phospho-ELk1(Ser383), Rabbit Polyclonal Antibody	Insight Biotechnology	Cat # bs-10154R
Anti-15-PGDH [EPR14332-19]	Abcam	Cat # ab187161
FITC Mouse Anti-Human HLA-DR,DP,DQ	Biolegend	Clone Tü39 Cat # 361706

Supplemental Table 7: Reagents

REAGENT	SOURCE	IDENTIFIER
K/BxN serum	PMID: 27158677	N/A
Liquid chromatography–grade water	Thermo Fisher	Cat # 51140
Ethanol, molecular grade	Sigma	Cat # E7023
Methanol, liquid chromatography–grade	Thermo Fisher	Cat # A452-1
Acetic acid, liquid chromatography–grade	Thermo Fisher	Cat # A35-500
Methyl formate, liquid chromatography–grade	Thermo Fisher	Cat # AC414345000
Hexane, liquid chromatography–grade	Thermo Fisher	Cat # H302-1
Indomethacin	Sigma	Cat # I7378
PBS	SLS, UK	Cat # LZBE17-516F
Deuterium-labelled 5S-HETE	Cayman Chemicals	Cat # 10007276
Deuterium-labelled leukotriene B_4	Cayman Chemicals	Cat #: 320110
Deuterium-labelled lipoxin A_4	Cayman Chemicals	Cat #: 10007737
Deuterium-labelled resolvin D2	Cayman Chemicals	Cat #: 11184
Deuterium-labelled prostaglandin E_2	Cayman Chemicals	Cat #: 314010
Arachidonic acid	Cayman Chemicals	Cat #: 10007268
Prostaglandin D_2	Cayman Chemicals	Cat #: 10007202
Prostaglandin E_2	Cayman Chemicals	Cat #: 10007211
Prostaglandin F_{2a}	Cayman Chemicals	Cat # 16010
Thromboxane B_2	Cayman Chemicals	Cat #: 10007237
Leukotriene B_4	Cayman Chemicals	Cat #: 10007240
Lipoxin A_4	Cayman Chemicals	Cat #: 10007271
Lipoxin B_4	Cayman Chemicals	Cat #: 90420
15-HETE	Cayman Chemicals	Cat #: 10007251
12-HETE	Cayman Chemicals	Cat #: 10007248
5-HETE	Cayman Chemicals	Cat #: 10007243
Δ6-trans-leukotriene B_4	Cayman Chemicals	Cat # 10007254
5S,12S-diHETE	In-house biogenic synthesis (J Dalli)	N/A
5S,15S-diHETE	Cayman Chemicals	Cat #: 35280
Δ6-trans,12-epi-LTB_4	Cayman Chemicals	Cat #: 35265
12-epi-LTB_4	Cayman Chemicals	Cat #: 20135
15-epi-LXA_4	Cayman Chemicals	Cat #: 90415
15-epi-LXB_4	Custom Synthesis (Dr Charles Serhan, Harvard Medical School)	N/A
Eicosapentanoic acid	Cayman Chemicals	Cat #: 90110
Resolvin E1	Cayman Chemicals	Cat #: 10007848
Resolvin E2	Cayman Chemicals	Cat #: 10007848
Resolvin E3	Cayman Chemicals	Cat #: 10007848
18-HEPE	Cayman Chemicals	Cat #: 32840
15-HEPE	Cayman Chemicals	Cat # 32700
12-HEPE	Cayman Chemicals	Cat #: 32540
5-HEPE	Cayman Chemicals	Cat #: 32200
Docosahexanoic acid	Cayman Chemicals	Cat #: 90310
Resolvin D1	Cayman Chemicals	Cat #: 10012554

Resolvin D2	Cayman Chemicals	Cat #: 10007279
Resolvin D3	Cayman Chemicals	Cat #: 13834
Resolvin D4	Custom Synthesis (Dr Charles Serhan, Harvard Medical School)	N/A
Resolvin D5	Cayman Chemicals	Cat #: 10007280
Resolvin D6	In-house biogenic synthesis (J Dalli)	N/A
17R-RvD1	Cayman Chemicals	Cat #: 13060
17R-RvD3	Custom Synthesis (Dr Charles Serhan, Harvard Medical School)	N/A
Maresin 1	Cayman Chemicals	Cat #: 10878
Maresin 2	Cayman Chemicals	Cat #: 16369
4S,14S-diHDHA	In-house biogenic synthesis (J Dalli)	N/A
7S,14S-diHDHA	In-house biogenic synthesis (J Dalli)	N/A
22-OH-Maresin 1	In-house biogenic synthesis (J Dalli)	N/A
Protectin D1	Custom Synthesis (Dr Charles Serhan, Harvard Medical School)	N/A
10S,17S-diHDHA	Custom Synthesis (Dr Charles Serhan, Harvard Medical School)	N/A
22-OH-Protectin D1	Custom Synthesis (Dr Trond V. Hansen, University of Oslo)	N/A
n-3 DPA	Cayman Chemicals	Item № 21907
$RvD1_{n\text{-}3\ DPA}$	In-house biogenic synthesis (J Dalli)	N/A
$RvD2_{n\text{-}3\ DPA}$	In-house biogenic synthesis (J Dalli)	N/A
$RvD5_{n\text{-}3\ DPA}$	In-house biogenic synthesis (J Dalli)	N/A
$Mar1_{n\text{-}3\ DPA}$	Custom Synthesis (Dr Trond V. Hansen, University of Oslo)	N/A
$PD1_{n\text{-}3\ DPA}$	Custom Synthesis (Dr Trond V. Hansen, University of Oslo)	N/A
10S,17S-diHDPA	In-house biogenic synthesis (J Dalli)	N/A
17R-PD1	Custom Synthesis (Dr Charles Serhan, Harvard Medical School)	N/A
13-series Resolvin 1	In-house biogenic synthesis (J Dalli)	N/A
13-series Resolvin 2	In-house biogenic synthesis (J Dalli)	N/A

13-series Resolvin 3	In-house biogenic synthesis (J Dalli)	N/A
13-series Resolvin 4	In-house biogenic synthesis (J Dalli)	N/A
INCB 024360	Axon Medchem	Cat # 1733
U0126	Tocris	Cat # 1144
Blood agar base	Oxoid/Thermo Fisher	Cat # CM0055
Horse blood, defibrinated	TCS Biosciences, UK	Cat # HB030
Brain heart infusion broth	Oxoid/Thermo Fisher	Cat # CM1135
Hemin	Sigma	Cat # H9039
β-Mercaptoethanol	Sigma	Cat # M6250
Lysing Matrix E	MP Biomedicals, UK	Cat # 116914100
Random Hexamers	Invitrogen/Thermo Fisher	Cat # SO142
Oligo(dT)20 Primers	Invitrogen/Thermo Fisher	Cat # 18418020
dNTP Mix (25 mM each)	Invitrogen/Thermo Fisher	Cat # R1121
RNaseOUT	Invitrogen/Thermo Fisher	Cat # 10777019
SuperScript III	Invitrogen/Thermo Fisher	Cat # 18080044
PowerUp SYBR Green Master Mix	Invitrogen/Thermo Fisher	Cat # A25776
Phenol:chloroform:isoamylalcohol, pH 8.0	Sigma	Cat # P2069
RPMI-1640	Sigma	Cat # R8758
DNaseI	Sigma	Cat # AMPD1-1KT
Collagenase D	Sigma	Cat # 11088866001
Penicillin-Streptomycin	Sigma	Cat # P4333
Fetal bovine serum	Thermo Fisher	Cat # 10500064
DPBS 0.0095M (PO4) without Ca and Mg	SLS, UK	Cat # LZBE17-512F
Bovine serum albumin	Sigma	Cat # A9418
LIVE/DEAD® Fixable Yellow Dead Cell Stain	Thermo Fisher	Cat # L34959
Merck-Millipore Periodic Acid Schiff (PAS) staining kit	VWR	Cat # 1.01646.0001
4% PFA solution	Affymetrix	Cat # 19943
123count eBeads	Thermo Fisher	Cat # 01-1234-42
Chloroform	Sigma	Cat # 02487
DAPI-containing ProLong Gold Antifade mountant	Thermo Fisher	Cat # P36935
10% neutral-buffered formalin	Sigma	Cat # HT501128
RNeasy Mini Kit	Qiagen, UK	Cat # 74104
DNeasy Blood and Tissue kit	Qiagen, UK	Cat # 69504
SiteClick™ Qdot™ 800 Antibody Labeling Kit	Thermo Fisher	Cat # S10455
APEX™ Alexa Fluor™ 647 Antibody Labeling Kit	Thermo Fisher	Cat # A10475
Melon™ Gel IgG Purification Kit	Thermo Fisher	Cat # 45212

Supplemental Figure legends:

Supplemental Figure 1: Inoculation of mice with *P. gingivalis* results in gut barrier breakdown in inflammatory arthritis. (**A**) Clinical arthritis scores were recorded over time after oral inoculation with *P. gingivalis* or *B. thetaiotaomicron* (10^9 CFU per mouse; Days -1, 1, 3) and injection of K/BxN serum (50µl per mouse, *i.p.*; Days 0, 2). Results are mean ± SEM for *n* = 4 mice per group per time point; two-way ANOVA followed by Bonferroni *post hoc* test, ****p*≤0.001, *****p*≤0.0001 K/BxN-injected +*P. gingivalis*-gavaged group (K/BxN + *P. gingivalis*) *versus* K/BxN-injected vehicle-gavaged group (K/BxN); $^{\#}p$≤0.05; $^{\#\#\#\#}p$≤0.0001 + *P. gingivalis* group *versus* K/BxN-injected *B. thetaiotaomicron*-gavaged control group (K/BxN + *B. thetaiotaomicron*). (**B**, **C**) Mice were inoculated with *P. gingivalis* (10^9 CFU per mouse) or given vehicle (PBS) on Days -1, 1, 3 and injected with K/BxN serum (50µl per mouse, *i.p.*; Days 0, 2). Tissues were harvested on day 8 after K/BxN administration and (**B**) bacterial translocation was assessed in intestinal sections using 16S FISH with a fluorescently labelled 16S rRNA gene probe (green) to visualize bacteria and DAPI (blue) to visualize host cells in colons of vehicle-gavaged control (K/BxN) and *P. gingivalis*-inoculated (K/BxN + *P. gingivalis*) mice. Dotted lines outline mucus layer, arrows denote bacterial invasion into the gut mucosa. NS Ctrl: non-specific scrambled oligonucleotide control probe, size bar = 25µm, inset: size bar = 12.5µm. Results are representative of n=4 mice per group from two independent experiments. (**C**) 16S rRNA gene levels were measured by 16S qPCR in mesenteric lymph nodes (MLN), spleens and livers of arthritic vehicle-gavaged mice (K/BxN) or *P. gingivalis*-inoculated mice (K/BxN + *P. gingivalis*) on Day 8 to assess breach of bacteria across the gut barrier. Results are mean ± SEM for *n* = 4 mice per group; unpaired t-test with Welch's correction, **p*≤0.05 *versus* K/BxN group.

Supplemental Figure 2: LC-MS/MS analysis of ALOX5 and ALOX15 activity in the small intestines of arthritogenic mice and lamina propria macrophages. (**A**) Arthritis was initiated as in Figure 1, small intestines harvested on day 8 post initiation, products were extracted and the concentrations of 17-HDPA and 7-HDPA were determined using LC-MS/MS. (**B**) Bone marrow derived macrophages were incubated with vehicle or immune complexes (37°C, 16h).

Products were extracted and the concentrations of 17-HDPA and 7-HDPA were determined using LC-MS/MS. Results mean ± sem. n = 4 mice per group.

Supplemental Fig.1

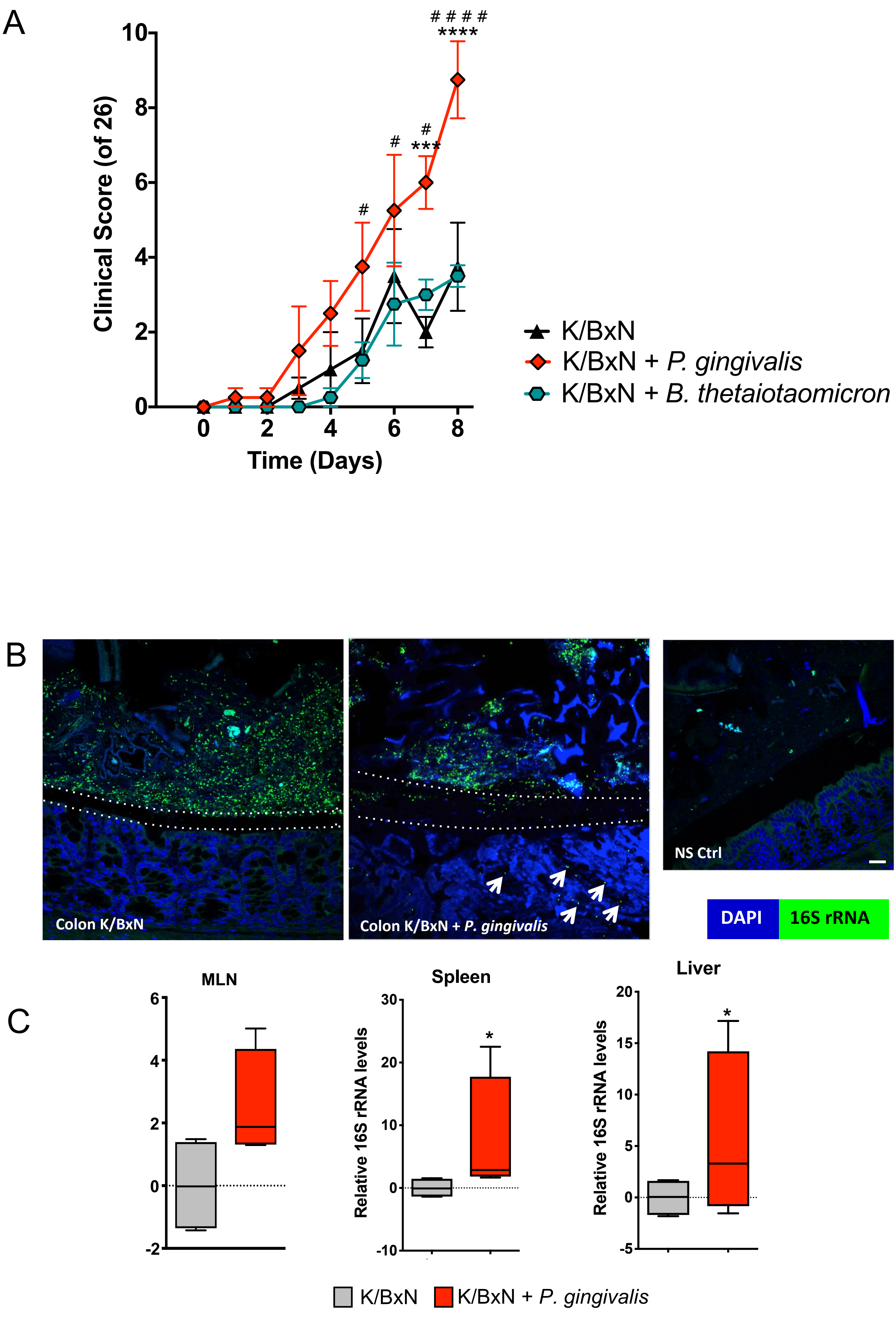

Supplemental Fig.2

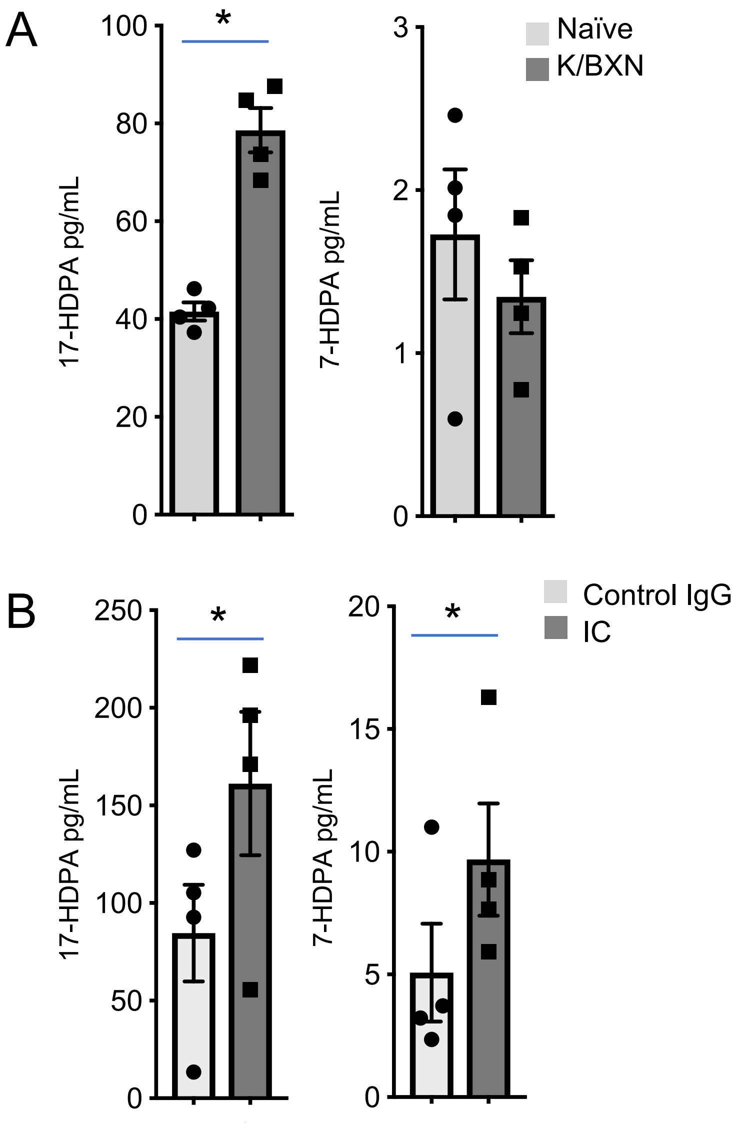

Supplement: Supplemental data [file jciinsight-4-125191-s007.pdf]
